# Identification of Phosphorylated Cyclin-Dependent Kinase 1 Associated with Colorectal Cancer Survival Using Label-Free Quantitative Analyses

**DOI:** 10.1371/journal.pone.0158844

**Published:** 2016-07-06

**Authors:** Peng-Chan Lin, Yi-Fang Yang, Yu-Chang Tyan, Eric S. L. Hsiao, Po-Chen Chu, Chung-Ta Lee, Jenq-Chang Lee, Yi-Ming Arthur Chen, Pao-Chi Liao

**Affiliations:** 1 Department of Internal Medicine, National Cheng Kung University Hospital, College of Medicine, National Cheng Kung University, Tainan, Taiwan; 2 Department of Environmental and Occupational Health, College of Medicine, National Cheng Kung University, Tainan, Taiwan; 3 Department of Medical Imaging and Radiological Sciences, Kaohsiung Medical University, Kaohsiung, Taiwan; 4 Center for Infectious Disease and Cancer Research, Kaohsiung Medical University, Kaohsiung, Taiwan; 5 Institute of Medical Science and Technology, National Sun Yat-Sen University, Kaohsiung, Taiwan; 6 Graduate Institute of Medicine, College of Medicine, Kaohsiung Medical University, Kaohsiung, Taiwan; 7 Institute of Basic Medical Sciences, College of Medicine, National Cheng Kung University, Tainan, Taiwan; 8 Department of Pathology, College of Medicine, National Cheng Kung University, Tainan, Taiwan; 9 Department of Surgery, College of Medicine, National Cheng Kung University, Tainan, Taiwan; University of Padova, ITALY

## Abstract

Colorectal cancer is the most common form of cancer in the world, and the five-year survival rate is estimated to be almost 90% in the early stages. Therefore, the identification of potential biomarkers to assess the prognosis of early stage colorectal cancer patients is critical for further clinical treatment. Dysregulated tyrosine phosphorylation has been found in several diseases that play a significant regulator of signaling in cellular pathways. In this study, this strategy was used to characterize the tyrosine phosphoproteome of colorectal cell lines with different progression abilities (SW480 and SW620). We identified a total of 280 phosphotyrosine (pTyr) peptides comprising 287 pTyr sites from 261 proteins. Label-free quantitative analysis revealed the differential level of a total of 103 pTyr peptides between SW480 and SW620 cells. We showed that cyclin-dependent kinase I (CDK1) pTyr15 level in SW480 cells was 3.3-fold greater than in SW620 cells, and these data corresponded with the label-free mass spectrometry-based proteomic quantification analysis. High level CDK1 pTyr15 was associated with prolonged disease-free survival for stage II colorectal cancer patients (n = 79). Taken together, our results suggest that the CDK1 pTyr15 protein is a potential indicator of the progression of colorectal cancer.

## Introduction

Colorectal cancer is a common disease and disease-specific mortality rate close to one-third in developed nations. [1]. According to an investigation by the American Cancer Society from 2004 to 2010, the five-year survival rate for early colorectal cancer stage I and II patients after diagnosis and treatment with surgery was 92% and 87%, respectively. In contrast, the five-year survival rates of colorectal cancer patients dramatically decreased to 53% and 11% for stage III and IV disease, respectively. The vast majority of colorectal cancer deaths are thought to be due to cancer metastasis and other cancer complications. The prognosis of colorectal cancer is affected by various features such as gender, age, and the quality of surgical intervention at the time of initial diagnosis. A number of colorectal tumor characteristics have also been applied to and evaluated for prognostic significance [2], such as lymphatic invasion, immunohistochemistry and plasma carcinoembryonic antigen (CEA) levels [3, 4]. This metastasis forms the basis of all the staging systems for this cancer have prognostic power compare with other signal feature at presentation [5–7]. Therefore, the identification of prognostic molecular biomarkers in surgical resection specimens is critical for helping us to predict disease-free survival of early stage colorectal cancer patients following curative surgery or for determining whether additional adjuvant therapies are needed.

Protein phosphorylation plays an important role in many biological processes, including cell proliferation, cell cycle regulation, and signaling pathways [8, 9]. Cells regulate phosphorylation via an enormous variety of protein kinase and phosphatases. Therefore, characterizing the phosphorylation status of proteins involved in complex cell signaling networks is critically important for understanding signal transduction within cells. Protein phosphorylation primarily occurs on three amino acids, which include serine, threonine, and tyrosine. Investigations of the phosphoproteome have estimated that the ratio of phosphorylation is 90%: 10%: <1% for phosphoserine (pSer), phosphothreonine (pThr), and phosphotyrosine (pTyr), respectively [10, 11]. Dysregulated tyrosine phosphorylation progressively increases in tumors during the progression and metastasis of colorectal carcinoma [12, 13] and lung cancer [14, 15]. Because of the relatively low abundance of tyrosine phosphorylation, many strategies and techniques have been specifically developed to separate and enrich tyrosine phosphopeptides from a sample. These enrichment methods for tyrosine phosphopeptides have included the following, antibody-based enrichment, which involves a number of phosphopeptide-specific antibodies [16–18]; metal oxide affinity chromatography, in which TiO_2_ enrichment is employed to enrich phosphopeptides [19–22]; and/or immobilized metal affinity chromatography (IMAC), in which metal ions (positive charges) are chelated to stationary beads and bind with phosphopeptides (negatively charges) in a mobile phase [23–25]. Currently, large-scale comparative phosphoproteomics studies based on mass spectrometry (MS) have emerged as significant tools. Such studies have supported and discriminated cellular protein phosphorylation in abnormalities leading to various phenotypes and diseases [14, 20, 26, 27].

In the present study, we employed biochemical characterization combined with a comparative phosphotyrosine proteome study of SW480 and SW620 cells to select potential prognosis biomarkers for colorectal cancer. Two colorectal cancer cell lines with different metastatic abilities, SW480 and SW620, were selected for this tyrosine phosphoproteome study. The SW480 and SW620 cells were derived from a primary colorectal adenocarcinoma and lymph node metastasis, respectively. Both cell lines were acquired at different stages of colon carcinoma in a single patient and therefore shared a genetic background, making it easier to study the genetic basis of their phenotypic differences [28]. In this study, we undertake to identify these protein targets that are at different levels in these two colorectal cell lines and to evaluate their prognostic value in colorectal cancer tissue.

## Materials and Methods

### Cell Lines

The colorectal cancer cell lines SW480 and SW620 were purchased from the Bioresource Collection and Research Center (BCRC), Hsinchu City, Taiwan. Briefly, the human colorectal adenocarcinoma cell lines SW480 and SW620 were maintained in Leibovitz's L-15 medium supplemented with 10% fetal bovine serum (FBS), 1% penicillin (Gibco, Life Technologies, Carlsbad, CA) and 1% streptomycin (Thermo) and were incubated at 37°C under 5% CO_2_.

### Western Blot Analysis

Ten micrograms of total protein from both the SW480 and SW620 cell lysates were individually separated on NuPAGE4-12%Bis-Tris gels (Life Technologies, Carlsbad, CA) using a Novex Mini-Cell system. The proteins were transferred to a PVDF membrane and the steps for transfer with PVDF membrane according to the iBlot’s protocol (Life Technologies). Following the PVDF membrane was blocked with 5% non-fat milk in 1XPBST. The PVDF membranes were separately probed with an anti-GAPDH antibody (GeneTex, San Antonio, TX), an anti-CDK1 (phospho Y15) antibody (Epitomics, clone EPR7875, cat. no ab133463, and dilution 1:1000) and an anti-phosphotyrosine-CDK2 (pTyr15) antibody (Epitomics, clone EPR2233Y, cat. no ab176146, and dilution 1:1000). The PVDF membranes were washed with 1XTBST (Tris-buffered saline and Tween-20) and incubated with the appropriate horseradish peroxidase (HRP) conjugated-secondary antibodies (Sigma-Aldrich) for 1 h., signals were developed using an enhanced chemiluminescence reagent (PerkinElmer, Waltham, MA). Following photographed using a UVP BioSpectrum imaging system (UVP, Upland, CA).

### Immunoaffinity Enrichment for pTyr Proteins

Prior to immunoaffinity enrichment for tyrosine phosphopeptides from the two prepared colorectal cancer cell lysates, 700 μg of extracted proteins were resuspended in 300 μL of radioimmunoprecipitation assay (RIPA) buffer (50 mM Tris-HCl, 150 mM NaCl, 1% NP-40, 0.5% sodium deoxycholate, 0.1% SDS) and reacted with 50 μL of agarose-immobilized pTyr-specific antibodies 4G10 (10 μg) (Millipore, Temecula, CA) and PT66 (10 μg) (Sigma-Aldrich, St. Louis, MO) mixed in a 1:1 (w/w) ratio [20, 29]. Before starting the incubation process, the antibody-agarose beads were washed five times by 200 μL of RIPA buffer. The prepared cell lysates were added to the antibody-agarose beads with overnight incubated at 4°C. After incubation, the sample was centrifuged for five min at 5000 x *g* at 4°C and washed three times with 500 μL RIPA buffer and two times with 1 mL ddH_2_O. Bound proteins were eluted four times using 50 μL of sample buffer (Invitrogen) supplemented with 400 mM dithiothreitol (Sigma-Aldrich) at room temperature.

### Stacking Gel Concentration and In-gel Digestion

To concentrate the proteins eluted from the immunoaffinity enrichment, we employed an SDS-PAGE gel composed of a 5% acrylamide stacking gel and a 20% acrylamide separating gel. After electrophoresis, the protein was concentrated at the border between the stacking gel and the separating gel. The gel was then stained with Coomassie brilliant blue for 1 h. The protein band was detained and excised in 1 mm^3^ pieces, which were then subjected to in-gel digestion. The gel pieces were washed two times with a 1:1 solution of acetonitrile (ACN) (J.T. Baker) and 25 mM ammonium bicarbonate. Subsequently, the protein mixtures were reduced and alkylated [20]. Trypsin digestion was performed with overnight incubated at 37°C, and the digested proteins were lyophilized for further analysis.

### TiO_2_ micro-column Enrichment and Alkaline Phosphatase Treatment

Sample before loading to the TiO_2_ micro-column, column was rinsed with 100 μL of 80% ACN (containing 2% trifluoroacetic acid (TFA)). Next step, the TiO_2_ micro-column was equilibrated with loading buffer (containing 65% ACN and 2% TFA with saturated glutamic acid (J.T. Baker)) for two min; this process was repeated three times. Add 185 μL of loading buffer to each sample (trypsin-digested samples (15 μL) from different cell lysates) and loaded into the TiO_2_ micro-column with a syringe pump (set at a flow rate of 10 μL/min). The micro-column was washed in the following order: (i) 20 μL sample loading buffer, (ii) 20 μL 65% ACN/0.5% TFA, and (iii) 20 μL of 65% ACN/0.1% TFA. To elute the bound peptides, add the 30 μL of 300 mM NH_4_OH/50% ACN to each column. An aliquot of the TiO_2_-eluted sample was added with 0.25 U of alkaline phosphatase (Roche, Applied Science, Mannheim, Germany) in 1×dephosphorylation buffer, pH 8.0 (Roche), at 37°C for 2 hr.

### LC-MS and Targeted LC-MS/MS Analyses

The peptide mixtures were analyzed by full-scan LC-MS on a nanoAcquity system (Waters, Milford, MA) coupled to an LTQ-Orbitrap spectrometer (Thermo Scientific) provided with a PicoView nanospray interface. Peptide mixtures were loaded into a 75-μm I.D. ×250-mm nanoACQUITY UPLC BEH130 column packed with C18 resin (Waters). The prepared peptide mixtures were separated by gradient buffer (buffer A: 0.1% formic acid in water; buffer B: 0.1% formic acid in ACN) with 10 to 35% solvent B for forty min, ensued by a sharp increase to 90% B in five min; then, a 300 nL/min flow rate was maintained for five minutes. The effluent from the HPLC column was electrosprayed directly into the MS. The LTQ-Orbitrap apparatus was ran in full-scan MS acquisition mode (*m*/*z* 300–2000). Targeted LC-MS/MS analysis was performed, and the possible phosphopeptide signals (*m*/*z* values) obtained from the iPhos process (see the next paragraph in the Methods section) were set in the parent mass list for LTQ-Orbitrap MS.

### Identification of Potential Phosphopeptide Signals by iPhos Software

The raw data generated by an LTQ-Orbitrap MS were converted to an open file representation mzXML format using an open-source program, msInspect [30], which was developed by the Computational Proteomics Laboratory (CPL); operational analysis was then conducted (http://proteomics.fhcre.org) to acquire peak information after peak detection, monoisotopic peak determination, and original mass calculation. Then, a TSV format file was produced for further iPhos software analysis. To determine the potential phosphopeptide signals, the iPhos program was used; a mass shift of -79.966 Da (or multiples of -79.966) due to dephosphorylation reactions was found in the detection by comparing the phosphopeptide signals and the corresponding dephosphorylated peptide signals. The iPhos program is an open source application written in Python 2.6 that operates in Windows (http://cosbi3.ee.ncku.edu.tw/iPhos/) [17, 31]. The iPhos program is designed to generate an inclusion list of potential phosphopeptide candidates for future targeted LC-MS/MS analysis.

### pTyr Protein Identification by Mascot Search

The raw data generated by the LTQ-Orbitrap MS were converted to an msm format using the Raw2msm program (MPI for Biochemistry, Martinsried, Germany). The MS/MS peak list was submitted to the Mascot search server (Version: 2.2, Matrix Science Ltd., London, UK) for a search of the Swiss-Prot human non-redundant database (Database: SwissProt; update: Feb 19, 2014, total 542503 sequences; Taxonomy: Homo sapiens (20271 sequences)) with 95% confidence level or greater. Up to two missed cleavages were allowed for the performance of trypsin-specific. Carboxyamidomethylation, deamidation, oxidation, and phosphorylation were allowed as variable post-translation modifications. The mass deviations for the precursor ions and fragment ions were all set to 20 ppm. A false discovery rate of ≤ 2.5% was calculated by the Mascot-integrated decoy database search. Finally, the following three criteria were used to assess the results: (i) the Mascot search result reported a pTyr modification, (ii) the pTyr peptide score was above 25, and (iii) the pTyr peptide rank was number one in the Mascot search results. Only results that met all of these criteria were identified as approved pTyr peptides in this study.

### Label-Free Quantitative Progenesis QI Analysis

Data from the 12 LC-MS runs were aligned by using Progenesis QI software (Nonlinear Dynamics, Newcastle upon Tyne, UK). One run was chosen as the alignment reference, and the other runs were automatically aligned to the reference according to their retention times. Each run was illustrated by a proprietary algorithm as a two-dimensional feature map as *m*/*z* value versus retention time. Features with fewer than three isotopes and only one charge were masked at this point and excluded from further analyses. The control and treated runs were then divided into two appropriate groups, and the raw abundances (areas under the peaks) of all the features were normalized. The features (features with charges of < +2 were masked and excluded from further analyses) were used to calculate a normalization factor to correct the experimental variation between the runs. The iPhos program added an extra function, which utilized the parameters *m*/*z* value, RT adjustment, and charge state linked up with qualitative identification and quantitative proteomics analysis in phosphoproteomics research.

### Specimens

Colorectal cancer tissue (79 samples) from patients diagnosed with early stage II colorectal cancer according to the tumor-node-metastasis (TNM) staging system and who had undergone curative surgery for primary colorectal cancer were retrospectively provided by the Tissue Bank of National Cheng Kung University Hospital from 1999 to 2007(http://tissuebank.med.ncku.edu.tw/tbs/). After curative surgery for colorectal cancer lesion(s), patients routinely received postoperative 5-fluorouracil-based adjuvant chemotherapy. Patients in this cohort were tracked for the interval between the diagnosis and the last contact (disease-free survival or cancer relapse or death). The median tracking time was 109 months (ranging from 74.5 to 120 months). All retrospectively collected clinic samples of the colorectal cancer patients were approved by and complied with the regulations of the Ethics Committee of National Cheng Kung University Hospital (Name of the IRB: The Use of Tyrosine Phosphoproteomics Technology for Identifying The Colorectal Cancer Biomarker Correlated with Early Recurrence; Reference Code: ER-100-294). All clinic participants were informed and gave their express agree to participate in this study. All clinic participants had to sign the consent forms before patient participated in this study.

### Immunohistochemistry

The immunohistochemistry (IHC) of tissue specimen of 79 of stage II colorectal cancer was examined in duplicate by sampling tumor tissue cores (4-μm-thick paraffin-embedded sections) from the Tissue Bank of National Cheng Kung University Hospital. The process were performed according to the Bond-Max automated IHC stain protocol (Leica Biosystems Newcastle Ltd.). IHC was performed using anti-CDK1 (phospho Y15) (Epitomics, clone EPR7875, cat. no ab133463, and dilution 1:250). Counterstaining was performed with hematoxylin. The IHC of each tissue specimen were evaluated under a light microscope (Zeiss Axio Imager A1, Jena, Germany) at 200× magnification.

### Statistical analysis

All data are expressed as the mean ± standard deviation (SD). CDK1 pTyr15 level and clinical and pathologic parameters were assessed by the chi-square test, Fisher's exact test or the Mann—Whitney *U*-test to determine the differences in non-parametric groups. ANOVA was used to determine the differences in parametric groups. Kaplan-Meier curves were used to assess the association of immunohistochemical reactivity of anti-CDK1 pTyr15 with disease-free survival. A *p*-value <0.05 based on two-tailed statistical analysis was considered statistically significant. All the above measures were calculated using SPSS software (Statistical Package for the Social Sciences, version18.0, Chicago, IL).

## Results and Discussion

### Strategy for Identifying the Tyrosine Phosphoproteome in Colorectal Cell Lines

**Fig 1:**
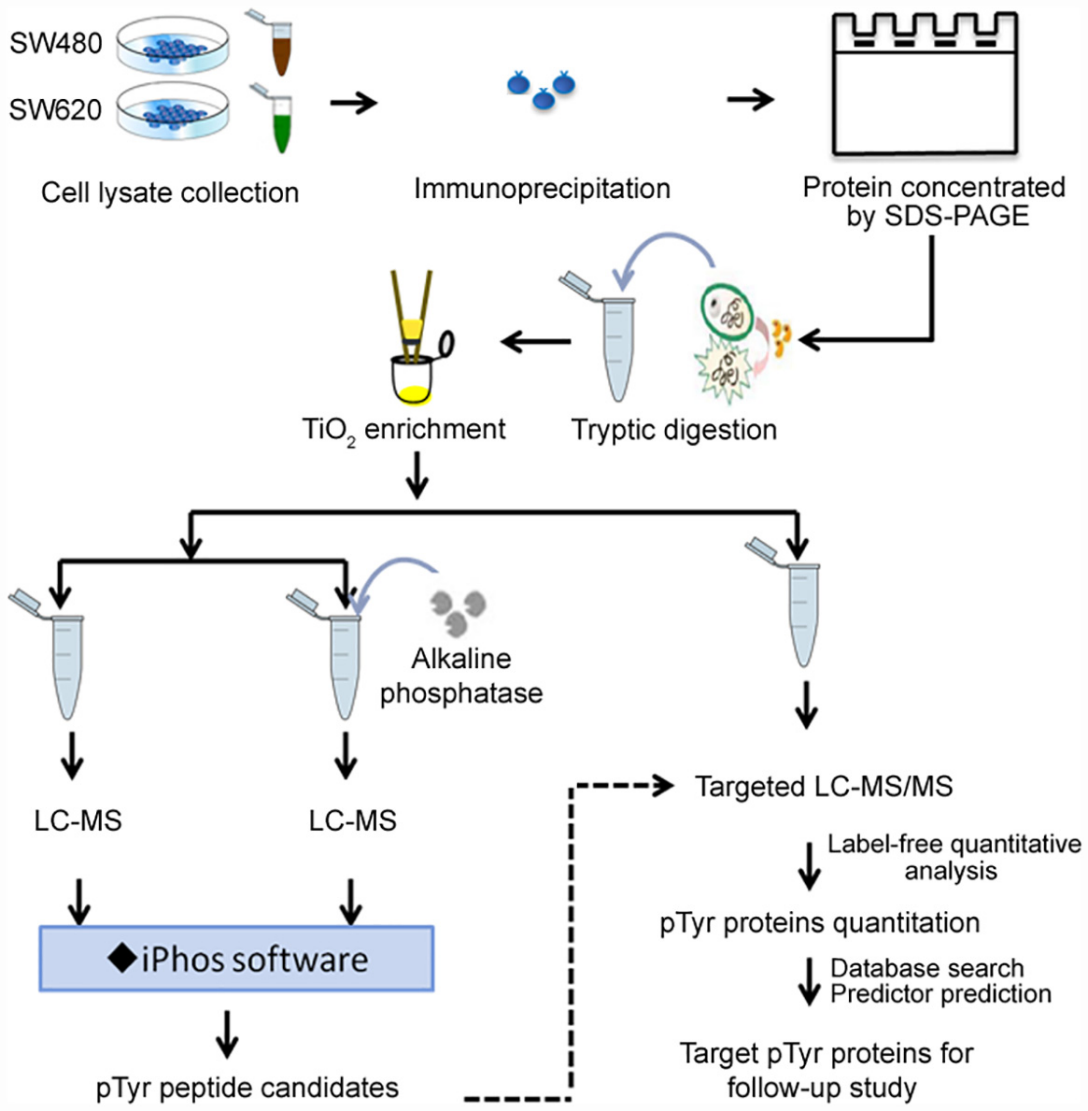
Experimental flowchart for the analysis of pTyr proteins in colorectal cancer cell lines.

To identify tyrosine-phosphorylated proteins associated with colorectal cancer progression, we analyzed proteins that were differentially expressed between the highly invasive SW620 colorectal cancer cells and their relatively noninvasive counterpart, SW480 cells (as shown in S1 Fig). Fig 1 represents the analytic strategy. Prior to LC-MS analysis, to diminish the potential for non-phosphoprotein interference and to increase the phosphotyrosine protein signals, the extracted proteins from the two colorectal cancer cells were separately reacted with the anti-pTyr antibodies 4G10 and PT66, mixed and applied for phosphotyrosine protein purification. After incubation, the eluents from the immunoprecipitated proteins were examined by SDS-PAGE. There was different levels of proteins with at least three protein bands labeled with 1 to 3 in the red rectangle between the SW480 and SW620 cells (S2 Fig). For further LC-MS analysis, eluted proteins from the immunoprecipitated purification of SW480 and SW620 cell lysates were individually run through an SDS-PAGE stacking gel to concentrate the protein and were then treated with in-gel tryptic digestion. An additional TiO_2_ micro-column after in-gel digestion was employed to remove non-phosphopeptides. From the TiO_2_ micro-column preparation for SW480 and SW620 cell samples, each prepared sample was aliquoted in pairs, with a phosphorylated portion (untreated with alkaline phosphatase) and dephosphorylated portion (treated with alkaline phosphatase). The phosphorylation and dephosphorylation portion pairs were separately analyzed by LC-MS. Two biological replicates of SW480 and SW620 samples (treated with and without alkaline phosphatase, respectively) were performed for LC-MS analysis (S3 Fig). Each biological replicate included three technical replicates, resulting a total of twenty-four analyses and providing information on reproducibility (n = 8x3 = 24) (S4 and S5 Figs). The detailed LC-MS analysis and results are described in the following section.

### Identification of Tyrosine-Phosphorylated Proteins in SW480 and SW620 Cells

**Fig 2:**
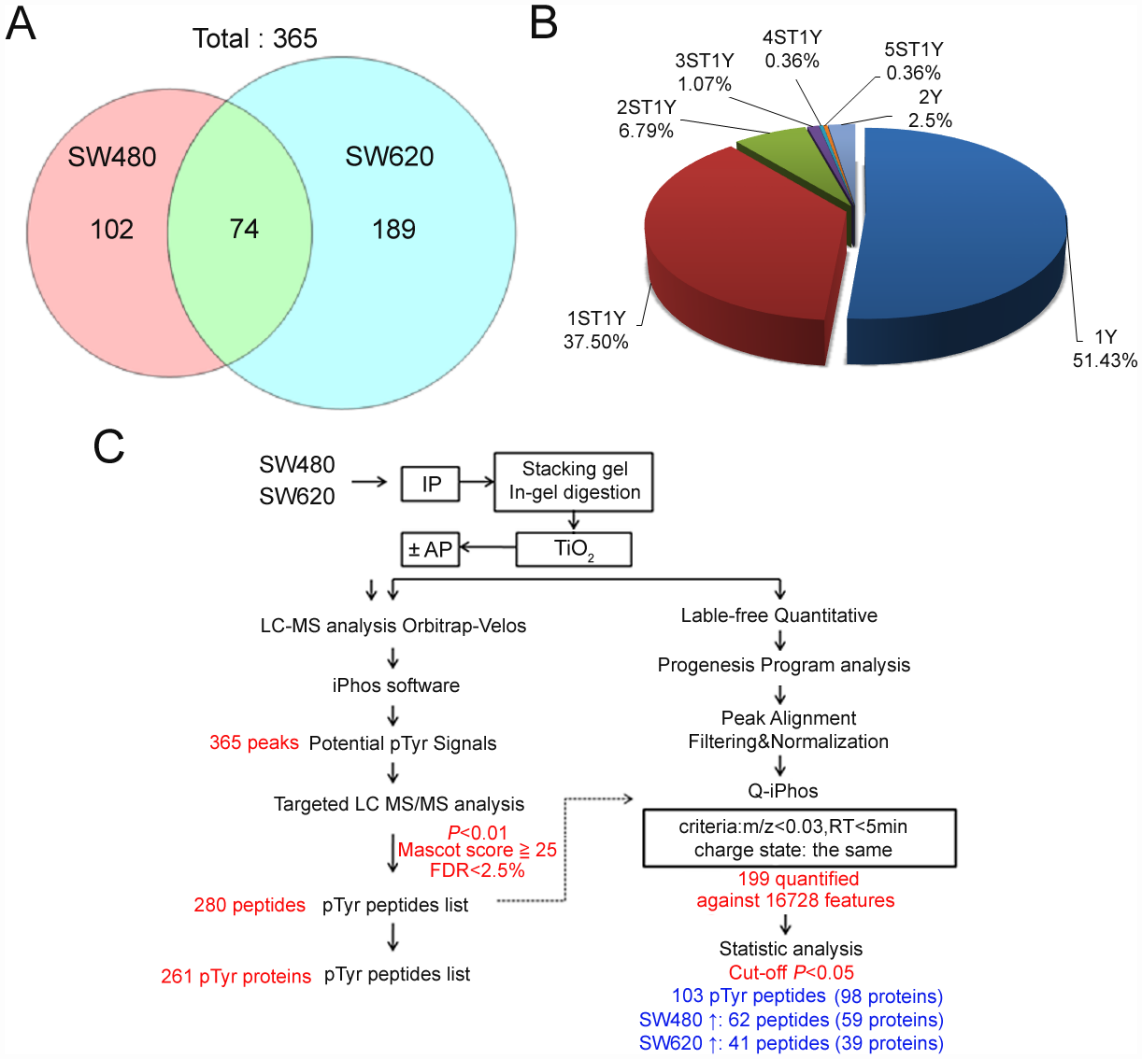
The identified phosphopeptide signals from LC-MS and LC-MS/MS analyses. (A) A total of 365 *m*/*z* signals that were identified as potential phosphopeptides from SW480 and SW620 cells were filtered using the iPhos program. There were 74 peptides that overlapped and presented in both cells. There were 102 and 189 potential phosphopeptides with differential level in the SW480 and SW620 cells, respectively. (B) A summary of single (n = 144, 51.4%), double (n = 105, 37.5%) and multiple (n = 31, 11.1%) phosphorylation sites from the identified phosphorylated peptides identified by the Mascot search. (C) A summary of the overall results through a flowchart of the label-free quantitative analysis.

After the LC-MS analysis, the results showed that 20,000 monoisotopic peaks were present in each LC-MS analysis. To produce a high yield of phosphopeptide signals for further targeted LC-MS/MS analysis in the iPhos analysis [32], the iPhos analytical parameters were set as follows, a maximum number of 1000 for the LC-MS dataset of phosphorylation and dephosphorylation pairs; a mass tolerance of 0.1 Da; a retention time tolerance after dephosphorylation within five min [17, 20, 33]; and a charge state of ≧2. The signals selected from the comparison with the signals for phosphorylation and dephosphorylation by iPhos showed that a total of 365 *m*/*z* signals were filtered from 6 LC-MS analyses of phosphorylation and dephos [17, 34]phorylation pairs for prepared SW480 and SW620 cells (Fig 2A). Furthermore, 74 signals of *m*/*z* were found in the integral multiple of 79.966 Da that shifted within the pairs of phosphorylated and dephosphorylated samples and that overlapped between the SW480 and SW620 cells, and 102 and 189 *m*/*z* signals were found in the integral multiple of 79.966 Da that shifted in the SW480 and SW620 cells, respectively. These 365 *m*/*z* signals generated by iPhos filtration were subjected to further targeted LC-MS/MS analysis. Based on the LC-MS investigation, we combined the comparative proteomics study with the alkaline phosphatase treatment/lack of treatment of SW480 and SW620 cells and demonstrated a possible increase in the efficiency of phosphopeptide identification after immunoprecipitation enrichment and iPhos signal filtration.

In the LC-MS/MS analysis, the MS/MS peak list was submitted and searched by the Mascot server. In the Mascot analysis, mass deviations for precursor ions and a false discovery rate of ≤ 2.5% were used. Finally, phosphotyrosine peptides were selected by following the Mascot search results reported for pTyr modification if the peptide score was greater than 25 and the peptide rank was 1 in the data identified from the LC-MS/MS analysis. A total of 280 pTyr peptides (approximately 76.7% (280/365) were identified by the targeted LC MS/MS analyses (S1 Table). After iPhos mass shifting analysis, the possibility of sequencing for low abundance pTyr peptides was increased [35, 36]. Further analysis revealed the distribution of single (n = 144, 51.4%), double (n = 105, 37.5%) and multiple (n = 31, 11.1%) phosphorylated peptides from the identified peptide (Fig 2B). In addition, 135 (47.0%, 135/287) pTyr sites had been reported in the PhosphoSitePlus database (PSP, http://www.phosphosite.org/) for protein phosphorylation sites [37]. A total of 103 (35.9%, 103/287) sites were predicted as potential pTyr sites from the phosphorylation site database (PHOSIDA, http://www.phosida.com/) [38, 39]. By combining these two results that 186 (64.8%) sites with either tyrosine phosphorylation sites (or predicted as possible substrates of tyrosine kinases).

### Tyrosine-Phosphorylated Proteins with Different Level in SW480 and SW620 Cells

In the relative label-free quantitative analysis of the identified pTyr peptides, the individual 6 replicate datasets from the targeted LC-MS analysis of SW480 and SW620 samples were aligned by using Progenesis QI software. Each feature with fewer than three isotopes and with charges < +2 were masked and excluded from further analyses. A total of 16,728 features were generated from the alignment of these targeted LC-MS/MS analysis datasets based on the drift of the retention time (< five minutes) and an *m*/*z* of < 0.03 Da. To examine the significance of the pTyr peptide levels for the prepared SW480 and SW620 samples using ANOVA, a total of 103 pTyr peptides from 98 proteins (*p*<0.05) were found, and 62 and 41 pTyr peptides had higher level in the SW480 and SW620 cells, respectively (Fig 2C). In addition, to confidently identify the pTyr peptides, the identified sequences were manually filtered with the following two criteria: (1) each identified pTyr peptide was more than three b or y ion signals near to or on the distributed pTyr site [20], and (2) the delta score in the Mascot scores of the peptides was more than 5 with different phosphorylated sites [40, 41]. By combining the targeted LC-MS/MS and Progenesis QI analyses, the following 10 pTyr proteins were found to have higher level in the SW480 cell line than in the SW620 cell line, poly (ADP-ribose) glycohydrolase (PARG), teratocarcinoma-derived growth factor I (TDGF1), HORMA domain-containing protein I (HORMAD1), phosphatidylinositol N-acetylglucosaminyltransferase subunit C (PIGC), neural cell adhesion molecule II (NCAM2), cyclin-dependent kinase 2 (CDK2), INO80 complex subunit D (INO80D), solute carrier organic anion transporter family member 2A1 (SLCO2A1), solute carrier family 12 member 1 (SLC12A1), and solute carrier family 12 member 1 (aMETTL19) (Table 1). In contrast, the following three pTyr proteins were found to have higher level in the SW620 cells than in the SW480 cells: fibroblast growth factor receptor III (FGFR3), putative RNA helicase Ski2w (SKI2W), and acyl-Coenzyme A dehydrogenase family member 8 isoform CRA_a (ACAD8) (Table 1).

**Table 1:** pTyr peptides with altered levels between SW480 and SW620 cells.

Gene name	Protein name	Peptide Score^a^	Ratio ^b^ (SW620/SW480)	*p*-value ^c^ (ANOVA-test)	Identified peptide sequence ^d^	pTyr site
**pTyr peptides with high levels in SW480**						
CDK2	Cyclin-dependent kinase 2	50	0.40	0.0275	IGEGpTpYGVVYK	Y15
PARG	Poly(ADP-ribose) glycohydrolase	49	0.06	8.90E-09	pYLDQFVPEK	Y832
INO80D	INO80 complex subunit D	39	0.23	0.0032	pYNpSQRCTNPIPK	Y57
SLC12A1	Solute carrier family 12 member 1	39	0.53	0.0008	MKPNTLVIGpYK	Y781
TDGF1	Teratocarcinoma-derived growth factor 1	34	0.29	0.0204	FSpYSVIWIMAISK	Y11
HORMAD1	HORMA domain-containing protein 1	32	0.16	1.97E-06	pYTNNGPLMDFISK	Y101
NCAM2	Neural cell adhesion molecule 2	33	0.10	0.0017	VAAVNGKGQGDpYSK	Y580
SLCO2A1	Solute carrier organic anion transporter family member 2A1	29	0.26	0.0287	pYLGLQMGYK	Y606
PIGC	Phosphatidylinositol N-acetylglucosaminyl transferase subunit C	28	0.35	0.0019	pYAQPVpTNpTK	Y2
METTL19	Probable tRNA (uracil-O(2)-)-methyltransferase	27	0.13	6.42E-06	MSNVpYQIQLSHSK	Y234
**pTyr peptides with high levels in SW620**						
FGFR3	Fibroblast growth factor receptor 3	49	1.87	0.0073	DGGEpYLCR	Y607
ACAD8	Acyl-Coenzyme A dehydrogenase family, member 8, isoform CRA_a	32	1.58	0.0152	QGDHpYILNGSK	Y56
SKI2W	Putative RNA helicase Ski2w	27	1.59	0.0447	RDIGFAASLpYTQ	Y1244

^a^ Peptide score derived from the mascot

^b^ Ratio derived from the normalized peak abundance from the SW620 sample divided by that of the SW480 sample, $Ratio=\;\bar{{\text{x}}_{620}}/\bar{{\text{x}}_{480}}$

^c^ p-value refers to the significance or the difference between SW480 and SW620 cells by using one-way ANOVA

^d^ Modified residues were underlined, indicating phosphorylation (STY), oxidation (M), carboxyamidomethylation (C), and deamidation (N,Q).

### Correlation between High level CDK1 pTyr15 and Good Prognosis in Stage II Colorectal Cancer Patients

**Fig 3:**
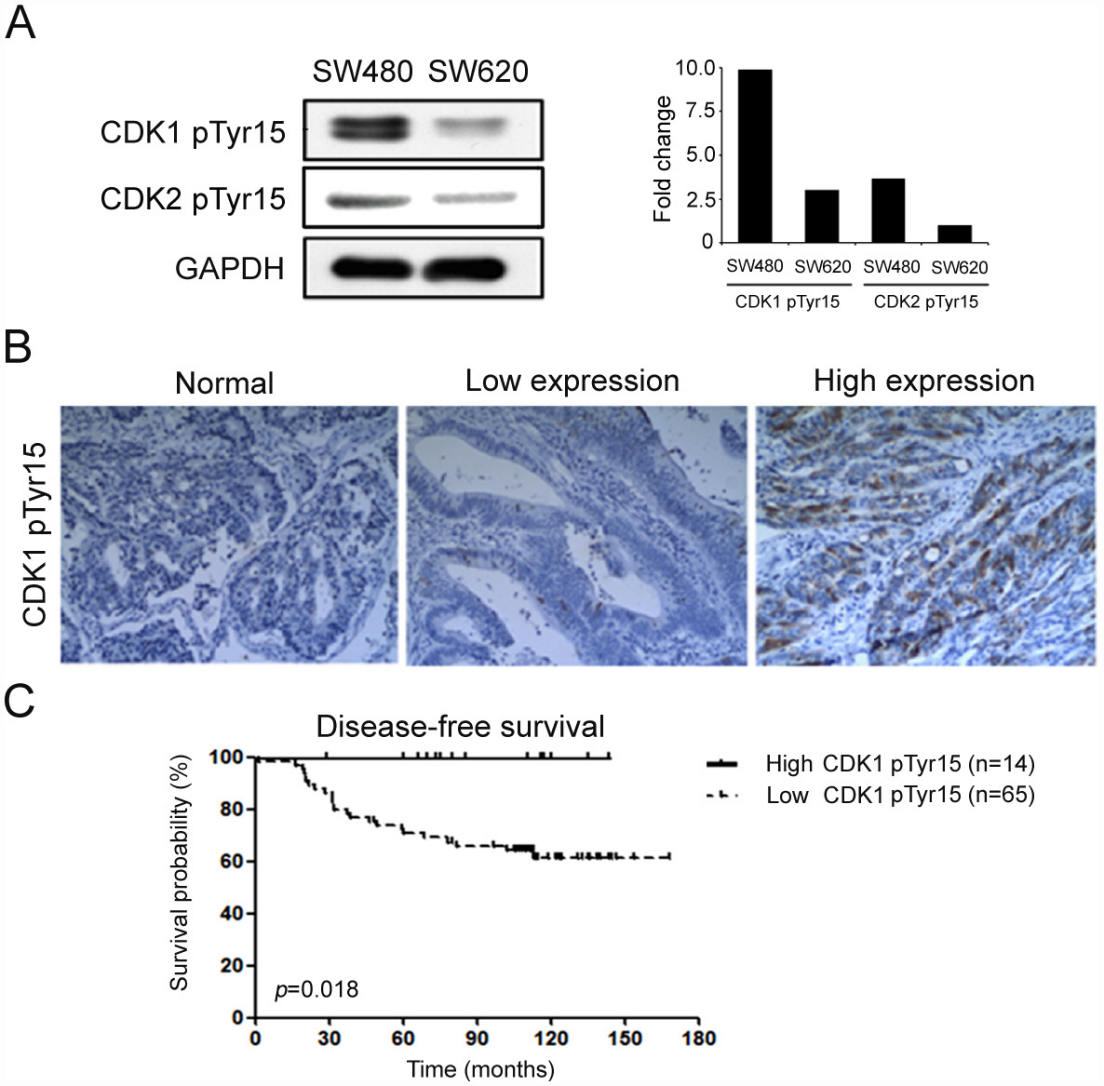
Differential expression of CDK1 pTyr15 in colorectal cancer cells and tissues. (A) Left panel, western blots of CDK1 pTyr15 and CDK2 pTyr15 expression in SW480 and SW620 cells. The relative intensity was quantified for CDK1 pTyr15 protein in SW480 and SW620 cells. Glyceraldehyde-3-phosphate dehydrogenase (GAPDH) was used as the loading control. Right panel, quantification of the western blot data is provided as the fold change. (B) Representative images of various CDK1 pTyr15 protein immunoreactivities in different samples of colorectal cancer tissues: left, the adjacent normal specimen staining; middle, the staining of the specimen was less than 50% total cancer cells; and right, the staining specimen was greater than 50% total cancer cells under a light microscope at 200× magnification. (C) Kaplan-Meier curves for disease-free survival for 79 stage II colorectal cancer patients according to CDK1 pTyr15 expression.

We selected CDK2 for further investigation based on the peptide score from label-free MS-based proteomics quantification (Table 1). First, the level of CDK2 pTyr15 in SW480 and SW620 cells was examined by western blot. The western blot analysis showed that the CDK2 pTyr15 had 3.6-fold higher level in the SW480 cells relative to the SW620 cells (Fig 3A). Additionally, level of the CDK family protein CDK1 was examined in SW480 and SW620 cells; CDK1 pTyr15 was uniquely up-regulated among other CDK family members in SW480 cells compared with SW620 cells (Fig 3A). Because CDK1 and CDK2 had the same phosphorylation site (^10^ IGEGTpYGVVYK^20^) and CDK1 pTyr15 was more highly expressed than CDK2 pTyr15 in SW480 cells, CDK1 was selected for further investigation. To examine the phosphorylation status of pTyr15 on CDK1 in human colorectal cancer tissues, immunoreactive intensities for CDK1 pTyr15 in surgical tumor tissues and adjacent normal tissues were compared. Representative results of the IHC analysis showed increased protein levels of CDK1 pTyr15 in tumor tissues compared with the adjacent normal, control tissues (Fig 3B). Based on the results of the IHC staining area, the tumor cells that could be counted into (1) low CDK1 pTyr15 level were named the ≦50% group of total cancer cells (n = 65), and (2) those with high CDK1 pTyr15 level were named the >50% group of total cancer cells (n = 14). After analyzing the IHC stained area, 50% staining of the total area of cancer cells was used as the cut-off value to evaluate stage II colorectal cancer patients. The results showed that CDK1 pTyr15 level was significantly associated with disease-free survival (*p* = 0.004) (Table 2). In addition, survival analysis showed that CDK1 pTyr15 level has the potential to be used as a biomarker to predict disease-free survival in stage II patients (Fig 3C). When CDK1 pTyr15 level was less than 50% of the staining area of the total cancer cells, the disease-free survival will decrease by approximately 30% after 60 months (*p* = 0.018). The results indicated that CDK1 pTyr15 level was a significant independent predictor of disease-free survival for colorectal cancer patients.

**Table 2:** Correlation between CDK1 pTyr15 expression and clinicopathological features in colorectal cancer.

CDK1 pTyr15 expression				
Characteristics	Number	High (>50%) (n = 14)	Low (<or = 50%) (n = 65)	*p* values
Gender				0.769^b^
Male	44	7	37	
Female	35	7	28	
Tumor status^a^				0.285^b^
T3	64	13	51	
T4	15	1	14	
Histological Differentiation				0.390^c^
Well	14	4	10	
Moderate	62	10	52	
Poor	3	0	3	
Original primary tumor				0.769^b^
Colon—Sigmoid	44	7	27	
Rectum	35	7	38	
Mucinous histology				0.213^b^
Yes	5	2	3	
No	74	12	62	
Disease-Free				0.004^b^
Yes	55	14	41	
No	24	0	24	

^***a***^ According the American Joint Committee on Colon and Rectum Cancer stage Manual, Seventh Edition (AJCC VII), the stages of T3 and T4 in Colon and rectum cancer were indicated tumor cells invades through the muscularis propria into pericolorectal tissues, and no regional lymph node metastasis; these T3 and T4 stages will be sorted into the overall stage IIA, IIB, or IIC for Colon and rectum cancer.

^***b***^ Analyzed with the Fisher's exact test

^***c***^ Analyzed with the Pearson Chi-Square (*X*^2^) test.

In this study, the purpose was to find prognostic biomarkers, to provide an accurate risk assessment for stage II colorectal cancer patients and to identify an aggressive therapy to follow surgery. Currently, a number of molecular biomarkers have been recommended, such as mutations in *KRAS* [42] and *CEA* [43]. Nevertheless, none of these candidate biomarkers have been clearly shown to be useful for the diagnosis or staging of patients with stage II colorectal cancer. Of the 10 highly expressed tyrosine phosphoproteins identified in the SW480 cells (Table 1), at least 4 candidates were able to anticipate cancer progression. PARG has been shown to participate in colon cancer cell growth and proliferation [44], TDGF1 is the founding member of the epidermal growth factor family and is overexpressed in the majority of human primary colorectal carcinomas and breast cancer [45], FGFR3 expression increases cell migration/invasion in colon cancer cells [46], and CDK1 has an essential role in control of the cell cycle and/or proliferation, and the dysregulation of CDK1 activity has been found in different human tumors [47, 48].

The CDK1 protein is a major player in a primary cell cycle checkpoint [49], whereby CDK1 interacts with cyclin B to form an active heterodimer that determines the timing of the G2 to M phase transition [49–51]. The cyclin B/CDK1 heterodimer is inactivated via the phosphorylation of CDK1 at two negative regulatory sites, tyrosine 15 (Tyr15) and threonine 14 (Thr14). Tyr15 is located in the ATP-binding site of CDK1 and inhibits phosphate transfer to a bound substrate, while phosphorylation of CDK1 Thr14 prevents ATP binding to CDK1. The inactivation of CDK1 via Tyr15 phosphorylation functions as a response to inhibit the initiation of mitosis in animal cells [52]. When cells in G2 phase proceed to M phase, CDK1 pTyr15 is dephosphorylated and activated by the protein phosphatase, cell division cycle 25 (CDC25) [53]. Phosphorylation of CDK1 by WEE1 leads to inactivation of CDK1, which prevents entry into the G2 to M transition [54–56]. However, recent reports have indicated WEE1 expression dose not markedly increased in CDK1 phosphorylation in human cancer [57]. Accumulating studies have shown that kinases as a target for cancer therapy use their driving action by overexpression, mutation and protein fusion [58]. Increased levels of WEE1 are observed in many different human cancers types, especially p53-deficient tumor [57, 59–62]. WEE1 is also overexpression in colon cancer [63]. WEE1 as target for anticancer therapy is considered to be potential therapeutic strategy when genomic instability with deficient p53 signaling and depend on WEE1 pathway for survival of mitosis [57]. PD0166285, a nonselective tyrosine kinase inhibitor of WEE1 to effect G2-abrogation when combined with irradiation that was used as an anticancer agent in experiments on various cancer cell lines [64–66]. Limitation of our study contain a relatively small sample size compared with other colon cancer studies. In this study, we propose that CDK1 pTyr15 (inactive form) acts as good prognostic biomarker in context of stage II colorectal cancer (n = 79). Comparing the report that specific activity of CDK1 was a predictor of tumor recurrence in stage II colon cancer from two patient cohorts (n = 254). The specific activity of CDK1 (active form) was significantly associated with worse distant metastasis rate and lower cause-specific survival [48]. The two studies described CDK1 differed in activation and inactivation, as well as associated poor prognosis and good prognosis in stage II colon cancer, respectively. Furthermore, CDK1 nuclear/cytoplasmic ratios in colon cancer tissue specimens predicts metastatic-free survival of affected patients (n = 164) [67]. We found that the low level of CDK1 pTyr15 was an indicator of the recurrence of colorectal cancer (Fig 3C). Although we cannot exclude possible contributions from Thr14 phosphorylation of CDK1 in colorectal cancer progression, this is the first study to evaluate that CDK1 pTyr15 predicted recurrence probabilities in stage II colorectal cancer.

## Conclusion

In summary, the signal intensity of pTyr peptides is considered to be relatively small during MS analysis due to a lower abundance, which impedes pTyr peptide identification. In this approach, based on the strategy of an initial specific immunoaffinity enrichment for the pTyr peptides and in-gel digestion, the subsequent TiO_2_ micro-column enrichment and combination with a home-developed iPhos program provided additional phosphopeptide enrichment and phosphopeptide signal identification, resulting in the identification of 280 pTyr peptides from the colorectal cancer cell lines SW480 and SW620 by LC-MS analysis. This phosphotyrosine proteome may provide a basic resource for studying the roles of protein tyrosine phosphorylation in colorectal cancer. From the label-free quantitative analysis, a total of 103 pTyr peptides from 98 proteins were identified, and 62 and 41 pTyr peptides were found to have higher differential expression in the SW480 and SW620 cells, respectively. High level of the candidate protein CDK1 pTyr15 in SW480 cells was validated by western blot. The results of the evaluation of clinical samples suggested that CDK1 pTyr15 is a potential indicator of the disease-free survival probability for stage II colorectal cancer patients. Other candidates, PARG and TDGF1, have been associated with cancer regulation; however, the functional role of the candidates HORMAD1, PIGC, NCAM2, INO80D, SLCO2A1, SLC12A1, and METTL19 was unclear in colorectal cancer. Further studies of their participation in the colorectal cancer process may be of great value.

## Supporting Information

S1 Fig Cell morphologies and biochemical characterization of SW480 and SW620 cell lines. (TIF)

S2 Fig Protein profiles of SW480 and SW620 cells. (TIF)

S3 Fig Samples preparation for LC-MS analysis. (TIF)

S4 Fig Reproducibility of LC-MS measurements for SW480 cell lines. (TIF)

S5 Fig Reproducibility of LC-MS measurements for SW620 cell lines. (TIF)

S1 Table Identified pTyr peptides in the SW480 and SW620 cell lines. (DOCX)
