# Supplementary figures and images for: Identification of Phosphorylated Cyclin-Dependent Kinase 1 Associated with Colorectal Cancer Survival Using Label-Free Quantitative Analyses
Source: PLoS One. 2016 Jul 6;11(7):e0158844. doi: 10.1371/journal.pone.0158844 (PMC4934865; doi:10.1371/journal.pone.0158844)

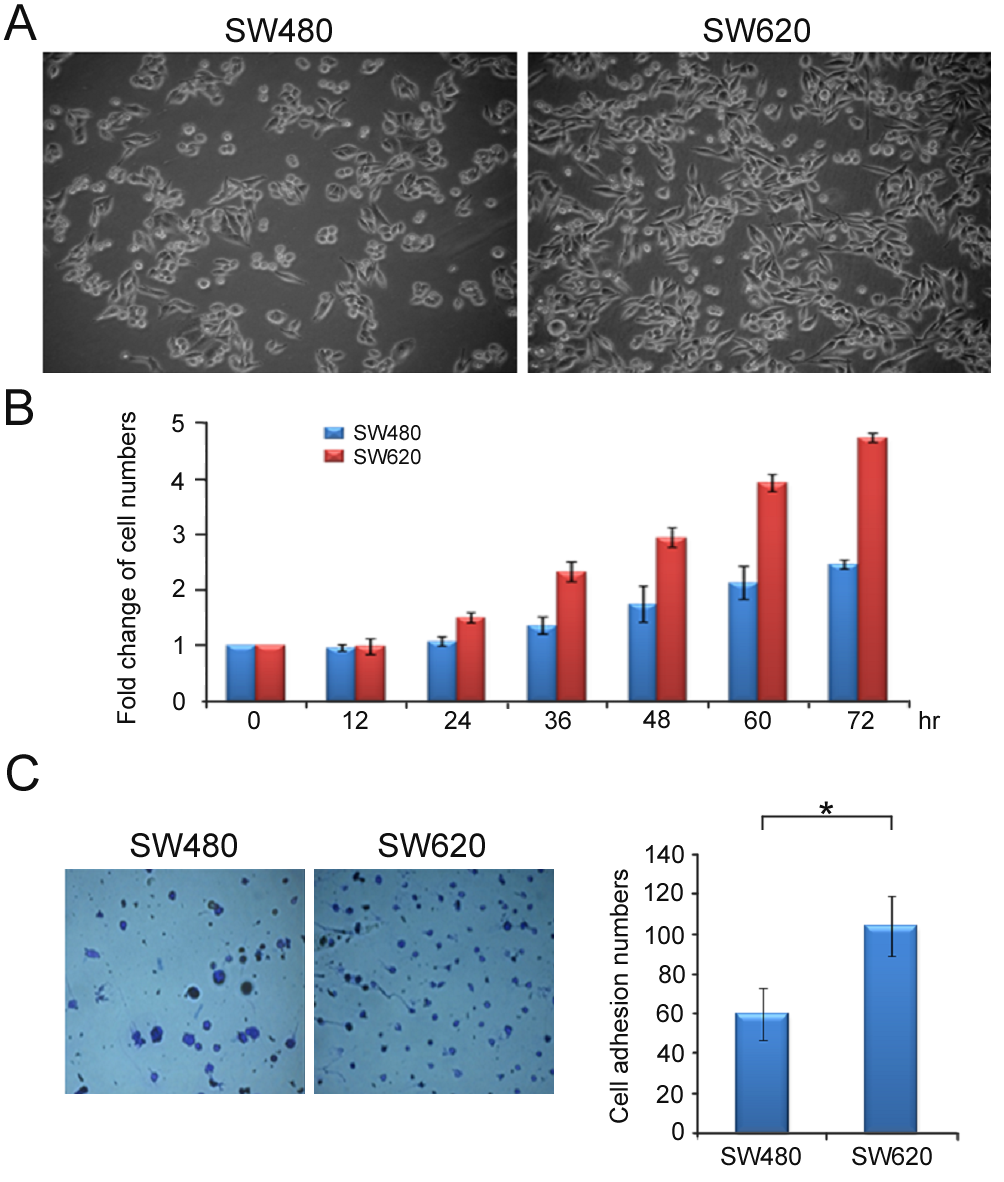

Supplement: S1 Fig — (TIF) [file pone.0158844.s001.tif]

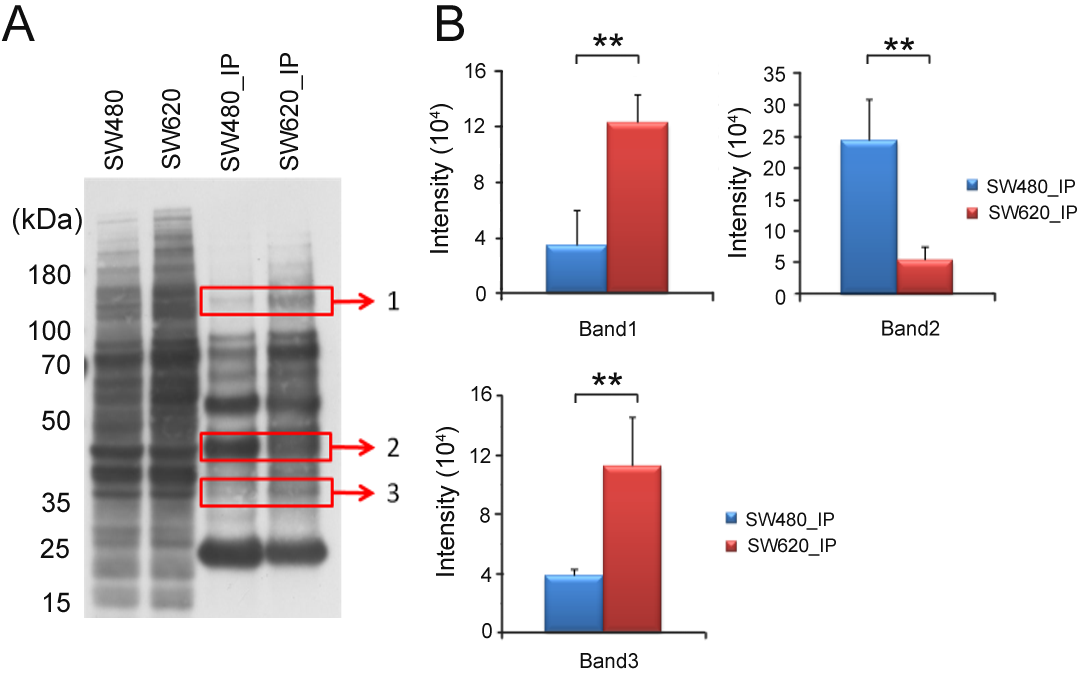

Supplement: S2 Fig — (TIF) [file pone.0158844.s002.tif]

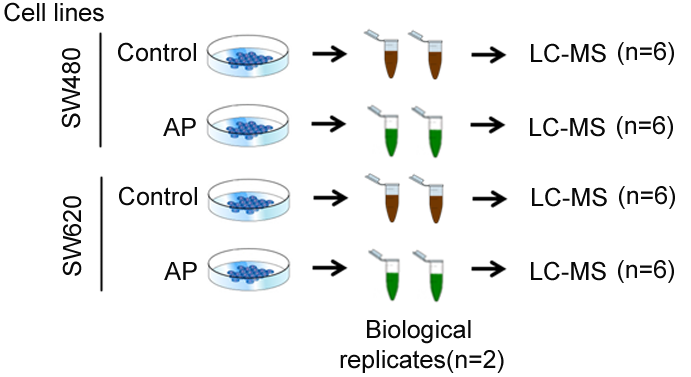

Supplement: S3 Fig — (TIF) [file pone.0158844.s003.tif]

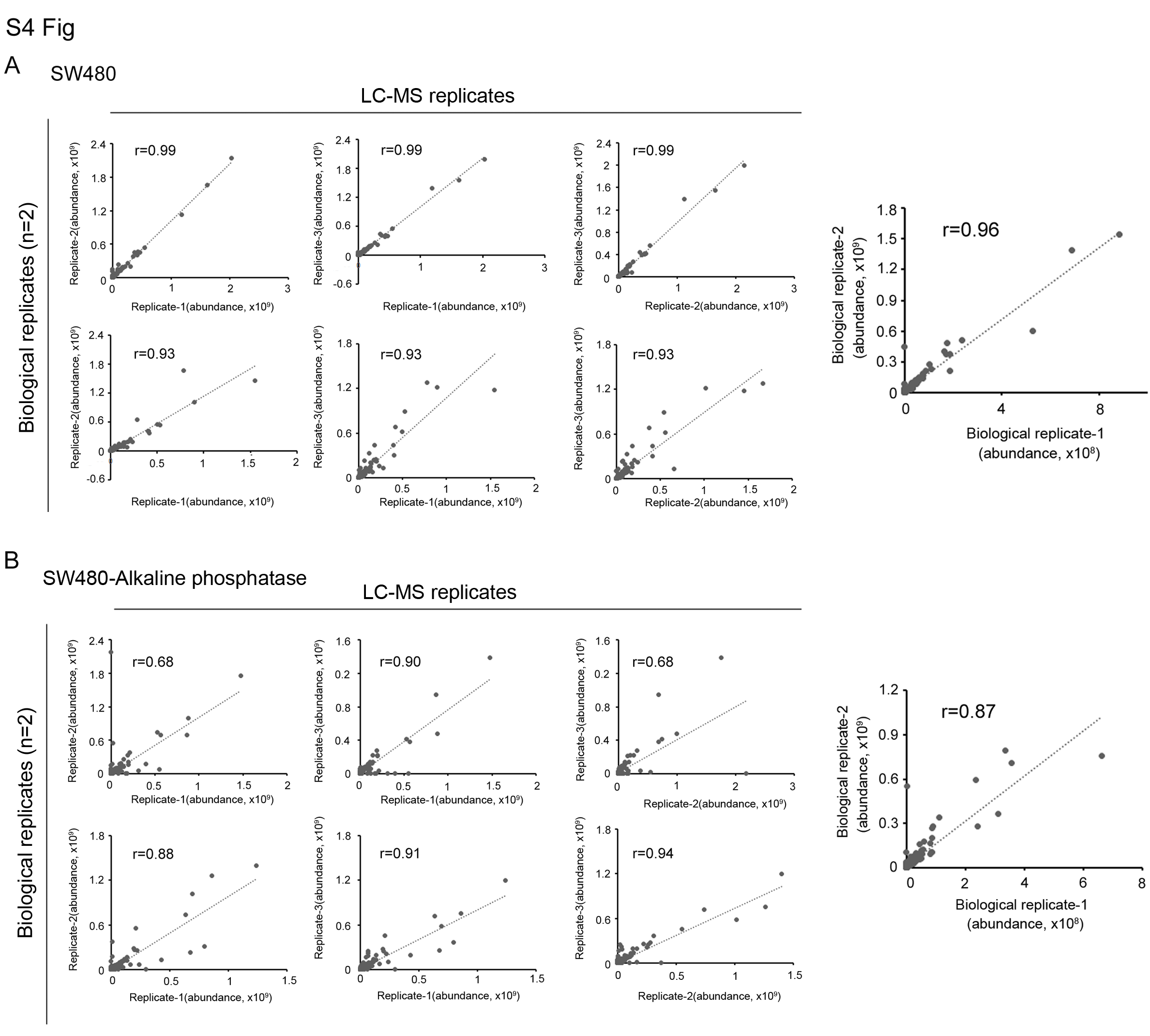

Supplement: S4 Fig — (TIF) [file pone.0158844.s004.tif]
