# Supplementary material for: Identification of Phosphorylated Cyclin-Dependent Kinase 1 Associated with Colorectal Cancer Survival Using Label-Free Quantitative Analyses
Source: PLoS One. 2016 Jul 6;11(7):e0158844. doi: 10.1371/journal.pone.0158844 (PMC4934865; doi:10.1371/journal.pone.0158844)
Supplement: S1 Table — (DOCX) [file pone.0158844.s006.docx]

**Supporting Information**

**Identification of Phosphorylated Cyclin-Dependent Kinase 1 Associated with Colorectal Cancer Survival Using Label-Free Quantitative Analyses**

Peng-Chan Lin ^1 ¶^ , Yi-Fang Yang^2¶^, Yu-Chang Tyan ^3,4,5,6^, Eric S.L. Hsiao^2^, Po-Chen Chu ^7^, Chung-Ta Lee ^8^ , Jenq-Chang Lee ^9^ , Yi-Ming Arthur Chen ^4,6^, Pao-Chi Liao ^2*^

* Correspondence should be addressed to Pao-Chi Liao,

E-mail: [liaopc@mail.ncku.edu.tw](mailto:liaopc@mail.ncku.edu.tw)

Inventory of all Supplemental Information

Supplemental materials and methods

Supplemental Data

S1 Figure Related to Figure 1

S2 Figure Related to Figure 2

S3 Figure Related to Figure 2

S4 Figure Related to Figure 2

S5 Figure Related to Figure 2

Supplementary S1Tables

S1 Table Related to Figure 2

**Supplemental Materials and Methods**

**Cell Proliferation and Cell Adhesion Assay**

Cell proliferation assays were performed for the SW480 and SW620 cancer cells, and a total of 1×10^3^ cells were individually seeded in each well of a 96-well plate. The cell proliferation within each group was checked every 12 h for 72 h. The results were acquired by culturing the cells at each checkpoint in three different wells and for three independent experiments, and the cell numbers were calculated from the average of six random fields under a light microscope at 200× magnification. Adhesion assays were performed and modified as previously described; 6-well plates were precoated with 30 μg/mL laminin in phosphate-buffered saline (PBS) at room temperature overnight. The coating buffer was then aspirated, and the wells were washed with PBS. The cultured SW480 and SW620 cells were suspended and changed to Leibovitz's L-15 media without serum, and 1×10^4^ cells were then seeded in each well of a 6-well plate and incubated for 1 h at 37°C. After incubation, the plates were aspirated and washed to remove non-adherent cells, and the adherent cells were fixed by 4% paraformaldehyde and stained with crystal violet solution. The cell adhesion results were counted under a light microscope (six randomly selected fields from three independent experiments at 200× magnification).

**Reproducibility of LC-MS Data Analysis**

In the reproducibility of LC-MS data analysis, the individual six replicates datasets from the LC-MS data of SW480 and SW620 (treated with and without alkaline phosphatase, respectively) samples were aligned by using Progenesis QI software. Plotting the abundance ratios from each two technical replicates [[1](#_ENREF_1), [2](#_ENREF_2)].

**References**

1. Krahmer N, Hilger M, Kory N, Wilfling F, Stoehr G, Mann M, et al. Protein correlation profiles identify lipid droplet proteins with high confidence. Molecular & cellular proteomics : MCP. 2013;12(5):1115-26. doi: 10.1074/mcp.M112.020230. PubMed PMID: 23319140; PubMed Central PMCID: PMC3650325.

2. de Graaf EL, Giansanti P, Altelaar AF, Heck AJ. Single-step enrichment by Ti4+-IMAC and label-free quantitation enables in-depth monitoring of phosphorylation dynamics with high reproducibility and temporal resolution. Molecular & cellular proteomics : MCP. 2014;13(9):2426-34. doi: 10.1074/mcp.O113.036608. PubMed PMID: 24850871; PubMed Central PMCID: PMC4159659.

**Supplemental Data**

**Supplementary Figure Legends**

**S1 Fig. Cell morphologies and biochemical characterization of SW480 and SW620 cell lines.** (A) Photomicrographs of SW480 and SW620 cells under a light microscope (at 100× magnification). (B) The cell proliferation of SW480 and SW620 cells. Cell growth within each group was measured every 12 h for 72 h. The quantification of cell numbers is expressed as the fold change (mean ± SD values) relative to 0 h in triplicate experiments. (C) Laminin-mediated cell adhesion. Quantification of the adherent cells is expressed as the mean ± SD values from triplicate experiments, and a Mann-Whitney *U*-test was performed (*p*<0.05).

**S2 Fig. Protein profiles of SW480 and SW620 cells.** (A) Total crude proteins of SW480 (lane 1) and SW620 cells (lane 2), and enriched proteins after immunoprecipitation from SW480 (lane 3) and SW620 cells (lane 4). Each sample was loaded for SDS-PAGE and stained with Coomassie blue. (B) Quantification of the intensities of different protein bands from the immunoaffinity enrichment for SW480 and SW620 cell lysates using ImageJ software.

**S3 Fig. Samples preparation for LC-MS analysis.** Two biological replicates of SW480 and SW620 samples (treated with and without alkaline phosphatase, respectively) were performed for LC-MS analysis. All samples included three technical replicates, resulting a total of twenty-four analyses and providing information on reproducibility.

**S4 Fig. Reproducibility of LC-MS measurements for SW480 cell lines.** (A) Abundance (LC-MS integration) comparisons for SW480 without AP LC-MS replicates. (B) Abundance (LC-MS integration) comparisons for SW480-treated with AP LC-MS replicates.

**S5 Fig. Reproducibility of LC-MS measurements for SW620 cell lines.** (A) Abundance (LC-MS integration) comparisons for SW620 without AP LC-MS replicates. (B) Abundance (LC-MS integration) comparisons for SW620-treated with AP LC-MS replicates.

**S1 Table. Identified pTyr peptides in the SW480 and SW620 cell lines.**

| Peptide index | SwissProt no. | Gene name | Protein name | Sequence***^a^*** | Charge state | m/z | Score ***^b^*** | Site | p-value ***^C^*** | Ratio ***^d^*** (SW620/SW480) | PSP | PHOSIDA |
| --- | --- | --- | --- | --- | --- | --- | --- | --- | --- | --- | --- | --- |
| 1 | Q8TD31 | CCHCR1 | Coiled-coil alpha-helical rod protein 1 | LpSpYAVRKVHTIR | 3 | 535.2362 | 31 | S465,Y466 | 0.9895 | 0.9546 |  |  |
| 2 | P20023 | CR2 | Complement receptor type 2 | pYSCpSGTFRLIGEK | 3 | 540.8951 | 27 | Y49,S52 | 0.9798 | 0.5186 | V |  |
| 3 | Q9UP83 | COG5 | Conserved oligomeric Golgi complex subunit 5 | EGKEFAPVpYPIMVQLLQK | 5 | 438.2188 | 28 | Y824 | 0.9707 | 0.7497 |  | V |
| 4 | Q9UI12 | ATP6V1H | V-type proton ATPase subunit H | pYNALLAVQK | 2 | 551.2753 | 33 | Y450 | 0.9514 | 1.1025 | V | V |
| 5 | O94811 | TPPP | Tubulin polymerization-promoting protein | HAGTpYDQKVQGGK | 2 | 734.8392 | 31 | Y211 | 0.9108 | 0.9552 |  |  |
| 6 | Q6P2P2 | PRMT10 | Putative protein arginine N-methyltransferase 10 | DEAAGpYFHK | 2 | 558.7576 | 27 | Y123 | 0.9080 | 0.6540 |  |  |
| Peptide index | SwissProt no. | Gene name | Protein name | Sequence***^a^*** | Charge state | m/z | Score ***^b^*** | Site | p-value ***^C^*** | Ratio ***^d^*** (SW620/SW480) | PSP | PHOSIDA |
| 7 | Q9HCI5 | MAGEE1 | Melanoma-associated antigen E1 | NHSYTLpYNRR | 2 | 702.8014 | 28 | Y815 | 0.8928 | 0.3340 | V |  |
| 8 | Q8NE09 | RGS22 | Regulator of G-protein signaling 22 | RELLpYPGpSpSK | 3 | 463.5400 | 31 | Y596,S599,S600 | 0.8853 | 0.9156 | V | V |
| 9 | Q8TBE9 | NANP | N-acylneuraminate-9-phosphatase | LAEECpYFLWK | 2 | 690.8100 | 30 | Y97 | 0.8579 | 0.9926 |  |  |
| 10 | Q32M45 | ANO4 | Anoctamin-4 | pYAEQMNVRMPFRR | 2 | 897.4129 | 31 | Y175 | 0.8336 | 0.8935 |  |  |
| 11 | Q6VBB8 | MLH1 | Mutant mismatch repair protein | QpYISEESTLSGQQ | 2 | 775.8218 | 34 | Y27 | 0.7943 | 0.3141 | V |  |
| 12 | Q96MN5 | TCEANC2 | Transcription elongation factor A N-terminal and central domain-containing protein 2 | KDpSGGKVpYK | 3 | 381.1801 | 26 | S18,Y24 | 0.7641 | 0.8139 |  |  |

| Peptide index | SwissProt no. | Gene name | Protein name | Sequence***^a^*** | Charge state | m/z | Score ***^b^*** | Site | p-value ***^C^*** | Ratio ***^d^*** (SW620/SW480) | PSP | PHOSIDA |
| --- | --- | --- | --- | --- | --- | --- | --- | --- | --- | --- | --- | --- |
| 13 | Q8NEP4 | C17orf47 | Uncharacterized protein C17orf47 | SVpSLQSGPGHpYAVPpTPR | 3 | 665.3586 | 26 | S72,Y80,T84 | 0.7103 | 1.0291 |  |  |
| 14 | Q96MU7 | YTHDC1 |  | pYVLQDARFFLIK | 3 | 531.5675 | 31 | Y350 | 0.7050 | 0.5622 |  |  |
| 15 | Q16831 | UPP1 | Uridine phosphorylase 1 | MCTLDFpYEGQGR | 2 | 749.8362 | 25 | Y214 | 0.6162 | 0.8946 | V | V |
| 16 | P49454 | CENPF | Centromere protein F | pYELVTELNDpSR | 2 | 750.2900 | 35 | Y1315,S1324 | 0.6139 | 0.9174 | V | V |
| 17 | O15480 | MAGEB3 | Melanoma-associated antigen B3 | KSpYKGANpSK | 3 | 381.1792 | 30 | Y83,S88 | 0.6079 | 0.7412 |  |  |
| 18 | Q7Z410 | TMPRSS9 | Transmembrane protease serine 9 | KCLISGWGpYLK | 3 | 468.8699 | 26 | Y333 | 0.5968 | 0.3582 |  |  |
| 19 | Q14656 | TMEM187 | Transmembrane protein 187 | GGAMGLGPRpYLK | 2 | 658.3098 | 26 | Y84 | 0.5774 | 0.9208 |  | V |

| Peptide index | SwissProt no. | Gene name | Protein name | Sequence***^a^*** | Charge state | m/z | Score ***^b^*** | Site | p-value ***^C^*** | Ratio ***^d^*** (SW620/SW480) | PSP | PHOSIDA |
| --- | --- | --- | --- | --- | --- | --- | --- | --- | --- | --- | --- | --- |
| 20 | P06239 | LCK |  | LIEDNEpYTAR | 2 | 652.2818 | 61 | Y394 | 0.5698 | 0.7828 | V |  |
| 21 | Q9UJU3 | ZFP112 | Zinc finger protein 112 homolog | SSpYLQAHQR | 2 | 585.2807 | 27 | Y567 | 0.5650 | 0.5661 |  | V |
| 22 | Q9Y251 | HPSE | Heparanase | pYLRLPYPFSNK | 2 | 739.3415 | 33 | Y463 | 0.5379 | 0.9650 | V | V |
| 23 | P08559 | PDHA1 | Pyruvate dehydrogenase E1 component subunit alpha, somatic form, mitochondrial | pYHGHSMSDPGVpSSRTR | 3 | 644.9404 | 25 | Y289,S300 | 0.5359 | 3.6502 | V |  |
| 24 | B4DI22 |  | cDNA FLJ51969, highly similar to Solute carrier family 12 member 1 | EMNpSGMAKKTGLApYK | 4 | 452.2080 | 31 | S543,Y553 | 0.4750 | 1.4315 |  |  |
| 25 | P78316 | NOP14 | Nucleolar protein 14 | TQTLLKEpYK | 2 | 602.7800 | 30 | Y74 | 0.4701 | 2.0621 |  |  |

| Peptide index | SwissProt no. | Gene name | | Protein name | | Sequence***^a^*** | | Charge state | | m/z | Score ***^b^*** | Site | p-value ***^C^*** | Ratio ***^d^*** (SW620/SW480) | PSP | | PHOSIDA | |
| --- | --- | --- | --- | --- | --- | --- | --- | --- | --- | --- | --- | --- | --- | --- | --- | --- | --- | --- |
| 26 | P17858 | PFKL | | 6-phosphofructokinase, liver type | | GGpSFENNWNIpYR | | 3 | | 540.5827 | 25 | S377,Y385 | 0.4609 | 2.4449 | V | | V | |
| 27 | Q9P0U3 | SENP1 | | Sentrin-specific protease 1 | | ELpTSVpYGSRAR | | 3 | | 466.9002 | 25 | T346,Y349 | 0.4542 | 0.9083 | V | | V | |
| 28 | Q6ZU52 | KIAA0408 | | Uncharacterized protein KIAA0408 | | AKIIDLpYHEK | | 3 | | 437.2260 | 28 | Y68 | 0.4448 | 0.2525 |  | |  | |
| 29 | B7Z6B4 | |  | | cDNA FLJ54967 | | SGpYLPpTMSIR | | 3 | 433.8629 | 25 | Y16,T19 | 0.4361 | 0.9792 |  | V | |  |
| 30 | P05165 | | PCCA | | Propionyl-CoA carboxylase alpha chain, mitochondrial | | MADALDNpYVIR | | 2 | 680.8567 | 38 | Y473 | 0.4236 | 2.4328 | V | V | |  |
| 31 | Q03393 | | PTS | | 6-pyruvoyl tetrahydrobiopterin synthase | | ISFSASHRLpYSK | | 2 | 737.8621 | 32 | Y27 | 0.4142 | 1.2053 | V | V | |  |
| 32 | Q8IX90 | | SKA3 | | Spindle and kinetochore-associated protein 3 | | NSIALVSTNpYPLSK | | 2 | 794.3416 | 26 | Y310 | 0.3977 | 0.6163 | V |  | |  |

| Peptide index | SwissProt no. | Gene name | Protein name | Sequence***^a^*** | Charge state | m/z | Score ***^b^*** | Site | p-value ***^C^*** | Ratio ***^d^*** (SW620/SW480) | PSP | PHOSIDA |
| --- | --- | --- | --- | --- | --- | --- | --- | --- | --- | --- | --- | --- |
| 33 | Q86UR5 | RIMS1 | Regulating synaptic membrane exocytosis protein 1 | pYRpSDPNLAR | 3 | 417.5297 | 27 | Y264,S266 | 0.3964 | 1.4545 | V |  |
| 34 | Q8IWN7 | RP1L1 | Retinitis pigmentosa 1-like 1 protein | QLpYpTTpSGKK | 2 | 633.2900 | 28 | Y197,T198,S200 | 0.3814 | 1.1466 |  |  |
| 35 | Q8IYW5 | RNF168 | E3 ubiquitin-protein ligase RNF168 | VPpYpSKETAVMPCGR | 3 | 585.2718 | 27 | Y239,S240 | 0.3809 | 1.7504 | V |  |
| 36 | Q6ZUB0 |  | FAM75-like protein FLJ43859 | SpSNMLpSMENVGNpYQGCpSQETAPK | 4 | 704.7933 | 26 | S758,S762,Y769773 | 0.3595 | 1.0701 |  |  |
| 37 | Q8TE73 | DNAH5 | Dynein heavy chain 5, axonemal | pYVpSMGQGQEVHAR | 2 | 811.3579 | 31 | Y4072,S4074 | 0.3397 | 0.3474 | V | V |
| 38 | Q13344 |  | Fus-like protein | SSGGGGGpSGGGpYNR | 3 | 443.8750 | 27 | S231,Y235 | 0.3332 | 0.8261 |  |  |

| Peptide index | SwissProt no. | Gene name | Protein name | Sequence***^a^*** | Charge state | m/z | Score ***^b^*** | Site | p-value ***^C^*** | Ratio ***^d^*** (SW620/SW480) | PSP | PHOSIDA |
| --- | --- | --- | --- | --- | --- | --- | --- | --- | --- | --- | --- | --- |
| 39 | B7Z996 |  | cDNA FLJ54254, highly similar to Brain mitochondrial carrier protein 1 | LpYRYIPpTIR | 3 | 452.5169 | 25 | Y121,T126 | 0.3292 | 0.6764 |  | V |
| 40 | Q8NHM5 | KDM2B | Lysine-specific demethylase 2B | FAGpYIEK | 2 | 453.7155 | 32 | Y229 | 0.3276 | 0.6567 | V | V |
| 41 | Q92616 | GCN1L1 | Translational activator GCN1 | pYLpSHSEFK | 2 | 585.2957 | 41 | Y252,S254 | 0.3248 | 2.7178 | V |  |
| 42 | Q9H4F1 | ST6GALNAC4 | Alpha-N-acetyl-neuraminyl-2,3-beta-galactosyl-1,3-N-acetyl-galactosaminide alpha-2,6-sialyltransferase | NYSHpYLQKAR | 2 | 679.8544 | 25 | Y136 | 0.3090 | 0.5750 |  |  |
| 43 | Q8N3X1 | FNBP4 | Formin-binding protein 4 | QpYEINATPK | 3 | 381.8600 | 29 | Y592 | 0.3080 | 1.2194 | V |  |
| 44 | Q14697 | GANAB | Neutral alpha-glucosidase AB | NLGLpYVKTR | 3 | 381.8608 | 38 | Y489 | 0.3080 | 1.2194 | V |  |

| Peptide index | SwissProt no. | Gene name | Protein name | Sequence***^a^*** | Charge state | m/z | Score ***^b^*** | Site | p-value ***^C^*** | Ratio ***^d^*** (SW620/SW480) | PSP | PHOSIDA |
| --- | --- | --- | --- | --- | --- | --- | --- | --- | --- | --- | --- | --- |
| 45 | P53350 | PLK1 | Serine/threonine-protein kinase PLK1 | pYARpTMVDK | 2 | 572.2794 | 32 | Y582,T585 | 0.2987 | 1.1949 | V |  |
| 46 | Q8WZ42 | TTN | Titin | EDVGHpYVVK | 2 | 562.8211 | 25 | Y10461 | 0.2968 | 0.0180 | V | V |
| 47 | Q15678 | PTPN14 | Tyrosine-protein phosphatase non-receptor type 14 | LEEGMVFTEpYEQIPK | 3 | 631.6528 | 36 | Y913 | 0.2887 | 1.2049 | V | V |
| 48 | P10114 | RAP2A | Ras-related protein Rap-2a | pYEKVPVILVGNK | 3 | 480.2318 | 27 | Y106 | 0.2756 | 0.7325 | V | V |
| 49 | D3DQB3 | SPOCK1 | Sparc/osteonectin, cwcv and kazal-like domains proteoglycan (Testican) 1, isoform CRA_a | MVCGSDGHSpYTSK | 3 | 489.8712 | 30 | Y10 | 0.2719 | 0.1540 |  |  |
| 50 | Q9P2D7 | DNAH1 | Dynein heavy chain 1, axonemal | YpTAGEINpYGGR | 3 | 454.2129 | 31 | T3940,Y3946 | 0.2542 | 0.8457 |  |  |

| Peptide index | SwissProt no. | Gene name | Protein name | Sequence***^a^*** | Charge state | m/z | Score ***^b^*** | Site | p-value ***^C^*** | Ratio ***^d^*** (SW620/SW480) | PSP | PHOSIDA |
| --- | --- | --- | --- | --- | --- | --- | --- | --- | --- | --- | --- | --- |
| 51 | Q13330 | MTA1 | Metastasis-associated protein MTA1 | HPpYLPINSAAIK | 3 | 468.8699 | 32 | Y500 | 0.2492 | 0.2664 |  | V |
| 52 | Q494X3 | ZNF404 | Zinc finger protein 404 | IHpTDLKPpYECNGCEK | 4 | 492.2336 | 34 | T182,Y187 | 0.2450 | 1.2433 | V |  |
| 53 | Q9NTI5 | PDS5B | Sister chromatid cohesion protein PDS5 homolog B | NLNKQApYDLAK | 3 | 453.2195 | 26 | Y215 | 0.2331 | 0.8376 | V |  |
| 54 | Q8IYJ3 | SYTL1 | Synaptotagmin-like protein 1 | pSpYLLPDKQSK | 2 | 670.3000 | 25 | S307,Y308 | 0.2267 | 0.6474 |  |  |
| 55 | Q02338 | BDH1 | D-beta-hydroxybutyrate dehydrogenase, mitochondrial | TpYApSAAEPVGSK | 2 | 670.3340 | 34 | Y46,S48 | 0.2267 | 0.6474 | V |  |
| 56 | Q6UXH1 | CRELD2 | Cysteine-rich with EGF-like domain protein 2 | TLpSKpYESSEIR | 2 | 736.8200 | 26 | S66,Y68 | 0.2076 | 3.4210 |  | V |
| 57 | Q9UM22 | EPDR1 | Mammalian ependymin-related protein 1 | MpYQQSSGRNpSR | 3 | 492.5816 | 35 | Y55,S63 | 0.2032 | 0.5938 |  |  |
| Peptide index | SwissProt no. | Gene name | Protein name | Sequence***^a^*** | Charge state | m/z | Score ***^b^*** | Site | p-value ***^C^*** | Ratio ***^d^*** (SW620/SW480) | PSP | PHOSIDA |
| 58 | B7Z5Z5 |  | cDNA FLJ53911, highly similar to PEX5-related protein | MpYQGHMQGKGSR | 3 | 492.5817 | 28 | Y2 | 0.2032 | 0.5938 |  |  |
| 59 | Q9UKY4 | POMT2 | Protein O-mannosyl-transferase 2 | QQQVTpTpYLHK | 2 | 703.2931 | 25 | T375,Y376 | 0.1817 | 0.3318 |  | V |
| 60 | Q9Y6X0 | SETBP1 | SET-binding protein | pYSGSGGDGGpSpTR | 2 | 670.3016 | 26 | Y1263,S1272,T1273 | 0.1771 | 1.8366 | V |  |
| 61 | Q8N394 | TMTC2 | Transmembrane and TPR repeat-containing protein 2 | AEANpYLR | 2 | 458.7455 | 36 | Y802 | 0.1725 | 2.0470 |  | V |
| 62 | Q7Z5V6 | PPP1R32 | Protein phosphatase 1 regulatory subunit 32 | FMTSEpYNSK | 3 | 401.1953 | 40 | Y179 | 0.1621 | 0.0206 |  |  |
| 63 | Q13029 | PRDM2 | PR domain zinc finger protein 2 | TCMpYQAQSRR | 2 | 690.3124 | 34 |  | 0.1586 | 1.7833 |  |  |
| 64 | P42702 | LIFR | Leukemia inhibitory factor receptor | SCpYQLEKTSIK | 2 | 690.3100 | 25 | Y91 | 0.1586 | 1.7833 | V |  |
| Peptide index | SwissProt no. | Gene name | Protein name | Sequence***^a^*** | Charge state | m/z | Score ***^b^*** | Site | p-value ***^C^*** | Ratio ***^d^*** (SW620/SW480) | PSP | PHOSIDA |
| 65 | Q9H0K6 | PUS7L | Pseudouridylate synthase 7 homolog-like protein | AITpYQAMVVRK | 3 | 459.8666 | 29 | Y345 | 0.1431 | 0.1534 |  | V |
| 66 | P20929 | NEB | Nebulin | NQENISSVLpYK | 3 | 459.8663 | 32 | Y6309 | 0.1431 | 0.1534 |  |  |
| 67 | Q8IUC8 | GALNT13 | Polypeptide N-acetylgalactosaminyltransferase 13 | pYYSLGEIR | 2 | 540.2715 | 37 | Y427 | 0.1420 | 1.2426 |  | V |
| 68 | P49840 | GSK3A | Glycogen synthase kinase-3 alpha | GEPNVSpYICSR | 2 | 681.7821 | 43 | Y279 | 0.1362 | 2.5317 | V |  |
| 69 | Q8IWX5 | SGPP2 | Sphingosine-1-phosphate phosphatase 2 | pYIGQVAKDVLK | 2 | 658.3349 | 30 | Y130 | 0.1300 | 0.7187 |  |  |
| 70 | P78527 | PRKDC | DNA-dependent protein kinase catalytic subunit | NTCTSVpYpTKDR | 3 | 483.2253 | 25 | Y115,T116 | 0.1231 | 0.1824 | V |  |
| 71 | Q13585 | GPR50 | Melatonin-related receptor | ASGHPKPHSRpSSpSApYR | 3 | 655.3315 | 29 | S392,S394,Y396 | 0.1230 | 1.7259 |  |  |

| Peptide index | SwissProt no. | Gene name | Protein name | Sequence***^a^*** | Charge state | m/z | Score ***^b^*** | Site | p-value ***^C^*** | Ratio ***^d^*** (SW620/SW480) | PSP | PHOSIDA |
| --- | --- | --- | --- | --- | --- | --- | --- | --- | --- | --- | --- | --- |
| 72 | Q86TH1 | ADAMTSL2 | ADAMTS-like protein 2 | HVpYCKpTpSDGR | 3 | 468.8545 | 28 | Y763,T766,S767 | 0.1097 | 0.2404 |  |  |
| 73 | Q9H0W8 | SMG9 | Protein SMG9 | KSSALAEpYSR | 2 | 596.2649 | 30 | Y515 | 0.1051 | 1.6961 | V | V |
| 74 | Q9UKN7 | MYO15A | Myosin-XV | DGDDYpYDR | 2 | 549.7300 | 26 | Y246 | 0.0957 | 1.2910 |  | V |
| 75 | Q8N780 |  | CDNA FLJ25943 fis, clone JTH10559 | CAPGpYpTGDR | 2 | 549.7416 | 27 | Y98,T99 | 0.0957 | 1.2910 | V |  |
| 76 | P31040 | SDHA | Succinate dehydrogenase [ubiquinone] flavoprotein subunit, mitochondrial | IRAKNTVVATGGpYGR | 3 | 548.8862 | 30 | Y259 | 0.0894 | 0.5466 | V |  |
| 77 | Q86YJ7 | ANKRD13B | Ankyrin repeat domain-containing protein 13B | GpYpSMMGGQR | 2 | 582.2822 | 31 | Y489,S490 | 0.0855 | 0.3400 |  | V |
| 78 | Q9H3A7 |  | HCG1983393 | KNpYYKpSPNLVR | 2 | 771.8490 | 34 | Y79,S82 | 0.0825 | 5.4597 |  |  |
| Peptide index | SwissProt no. | Gene name | Protein name | Sequence***^a^*** | Charge state | m/z | Score ***^b^*** | Site | p-value ***^C^*** | Ratio ***^d^*** (SW620/SW480) | PSP | PHOSIDA |
| 79 | Q9H3A7 | hCG_1983393 | HCG1983393 | KNpYpYKSPNLVR | 2 | 771.8500 | 32 | Y79,Y80 | 0.0825 | 5.4597 |  |  |
| 80 | Q59GV3 |  | SWI/SNF-related matrix-associated actin-dependent regulator of chromatin c2 isoform b variant | KGPSTPpYpTKSK | 3 | 451.5423 | 30 | Y336,T337 | 0.0791 | 1.5350 | V |  |
| 81 | O43451 | MGAM | Maltase-glucoamylase, intestinal | pYEYGpTLDNMR | 3 | 480.2318 | 26 | Y385,T389 | 0.0790 | 0.6447 |  | V |
| 82 | Q14457 | BECN1 | Beclin-1 | pYAQTQLDKLK | 2 | 644.7757 | 38 | Y256 | 0.0698 | 1.3367 |  |  |
| 83 | Q4FZB7 | SUV420H1 | Histone-lysine N-methyltransferase SUV420H1 | pYSpSEQNGAPIVATK | 2 | 812.3530 | 30 | Y203,S205 | 0.0669 | 0.6012 | V |  |
| 84 | Q8IZ52 | CHPF | Chondroitin sulfate synthase 2 | MEpYTLDLQLEALpTPQGGR | 3 | 738.3389 | 30 | Y463,T473 | 0.0661 | 0.6658 | V |  |

| Peptide index | SwissProt no. | Gene name | Protein name | Sequence***^a^*** | Charge state | m/z | Score ***^b^*** | Site | p-value ***^C^*** | Ratio ***^d^*** (SW620/SW480) | PSP | PHOSIDA |
| --- | --- | --- | --- | --- | --- | --- | --- | --- | --- | --- | --- | --- |
| 85 | Q14204 | DYNC1H1 | Cytoplasmic dynein 1 heavy chain 1 | DAATIMQPpYFpTSNGLVpTK | 3 | 738.3392 | 27 | Y2426,S2428,T2434 | 0.0661 | 0.6658 | V |  |
| 86 | Q9UHD2 | TBK1 | Serine/threonine-protein kinase TBK1 | EPLDTIGLIpYEK | 3 | 490.9007 | 29 | Y394 | 0.0639 | 8.2761 | V | V |
| 87 | Q8NA61 | SPERT | Spermatid-associated protein | MApSQHpSpYPLNR | 3 | 515.6108 | 34 | S86,S89,Y90 | 0.0633 | 11.6839 |  |  |
| 88 | Q8N2G4 | LYPD1 | Ly6/PLAUR domain-containing protein 1 | EVMEQpSAGIMpYRK | 3 | 573.2844 | 27 | S62,Y67 | 0.0629 | 4.1880 |  |  |
| 89 | Q29RF7 | PDS5A | Sister chromatid cohesion protein PDS5 homolog A | CLpYYLpYASLDPNAVK | 3 | 631.9479 | 25 | Y488,Y491 | 0.0595 | 1.5845 |  |  |
| 90 | O76000 | OR2B3 | Putative olfactory receptor 2B3 | MVSLFpYGIITS | 2 | 655.3228 | 27 | Y278 | 0.0594 | 0.3502 |  | V |
| 91 | P24941 | CDK2 | Cyclin-dependent kinase 2 | IGEGpTpYGVVYK | 2 | 673.2775 | 44 | T14,Y15 | 0.0594 | 2.1520 | V | V |
| Peptide index | SwissProt no. | Gene name | Protein name | Sequence***^a^*** | Charge state | m/z | Score ***^b^*** | Site | p-value ***^C^*** | Ratio ***^d^*** (SW620/SW480) | PSP | PHOSIDA |
| 92 | Q96M11 | HYLS1 | Hydrolethalus syndrome protein 1 | EAQpSIQYDPpYSK | 2 | 795.3393 | 28 | S45,Y51 | 0.0588 | 0.3789 | V |  |
| 93 | Q9BYG3 | MKI67IP | MKI67 FHA domain-interacting nucleolar phosphoprotein | QPpSpYPSVKR | 3 | 408.1864 | 28 | S142,Y143 | 0.0566 | 3.7087 | V |  |
| 94 | Q9H4L5 | OSBPL3 | Oxysterol-binding protein-related protein 3 | pYAKSQTDIER | 3 | 431.2140 | 30 | Y83 | 0.0546 | 0.6167 | V |  |
| 95 | Q8WZ42 | TTN | Titin | SSKpYISSLEILR | 3 | 492.8956 | 33 | Y4436 | 0.0514 | 2.3345 |  | V |
| 96 | Q9UJV9 | DDX41 | Probable ATP-dependent RNA helicase DDX41 | MVpYLLECLQK | 3 | 460.1986 | 26 | 423 | 0.0507 | 0.5308 |  |  |
| 97 | Q9NZN5 | ARHGEF12 | Rho guanine nucleotide exchange factor 12 | LQLLQEDpYNR | 2 | 686.3158 | 28 | Y227 | 0.0497 | 1.6632 | V | V |
| 98 | Q99533 | LAMA3 | Alpha3A | CAPGpYLGIPR | 2 | 563.2675 | 31 | Y153 | 0.0469 | 3.0685 | V | V |

| Peptide index | SwissProt no. | Gene name | Protein name | Sequence***^a^*** | Charge state | m/z | Score ***^b^*** | Site | p-value ***^C^*** | Ratio ***^d^*** (SW620/SW480) | PSP | PHOSIDA |
| --- | --- | --- | --- | --- | --- | --- | --- | --- | --- | --- | --- | --- |
| 99 | O76046 | SKI2W | Putative RNA helicase Ski2w | RDIGFAASLpYTQ | 3 | 474.8775 | 28 | Y1244 | 0.0447 | 1.5853 |  |  |
| 100 | E9PCU7 | PDCD2 | Uncharacterized protein | EDpYpSEIIGSMGK | 2 | 752.3587 | 26 | Y212,S213 | 0.0429 | 2.0815 | V | V |
| 101 | Q04446 | GBE1 | 1,4-alpha-glucan-branching enzyme | WELpYIPPK | 2 | 563.2460 | 26 | Y125 | 0.0410 | 0.4025 | V | V |
| 102 | P50479 | PDLIM4 | PDZ and LIM domain protein 4 | pYLQGMLEAGEGGDWPGPGGPRNLKPTApSK | 5 | 632.6638 | 28 | Y213,S240 | 0.0311 | 2.3246 | V |  |
| 103 | Q92560 | BAP1 | Ubiquitin carboxyl-terminal hydrolase BAP1 | GVQVEEIpYDLQSK | 2 | 795.3389 | 28 | Y33 | 0.0306 | 0.3781 | V | V |
| 104 | Q8NDV3 | SMC1B | Structural maintenance of chromosomes protein 1B | pYQSLLEELKMNK | 3 | 531.2555 | 26 | Y209 | 0.0288 | 0.5561 |  | V |
| 105 | Q92959 | SLCO2A1 | Solute carrier organic anion transporter family member 2A1 | pYLGLQMGYK | 2 | 584.7600 | 29 | Y606 | 0.0287 | 0.2566 |  | V |

| Peptide index | SwissProt no. | Gene name | Protein name | Sequence***^a^*** | Charge state | m/z | Score ***^b^*** | Site | p-value ***^C^*** | Ratio ***^d^*** (SW620/SW480) | PSP | PHOSIDA |
| --- | --- | --- | --- | --- | --- | --- | --- | --- | --- | --- | --- | --- |
| 106 | P41227 | NAA10 | N-alpha-acetyltransferase 10 | pYVpSLHVR | 2 | 517.2254 | 26 | Y106,S108 | 0.0279 | 4.1267 | V | V |
| 107 | P24941 | CDK2 | Cyclin-dependent kinase 2 | IGEGTpYGVVYK | 2 | 632.8000 | 50 | Y15 | 0.0275 | 0.4034 | V | V |
| 108 | Q9NZC4 | EHF | ETS homologous factor | KNNSpSMTpYEK | 2 | 681.7900 | 29 | S257,Y260 | 0.0218 | 0.1610 |  | V |
| 109 | Q8IZT6 | ASPM | Abnormal spindle-like microcephaly-associated protein | CRAYpYQGK | 2 | 563.2459 | 29 | Y2154 | 0.0216 | 0.3823 | V |  |
| 110 | Q2M2H8 |  | Putative maltase-glucoamylase-like protein LOC93432 | pYLIIMNPGISK | 2 | 665.3089 | 26 | Y402 | 0.0210 | 2.7570 |  | V |
| 111 | P13385 | TDGF1 | Teratocarcinoma-derived growth factor 1 | FSpYSVIWIMAISK | 2 | 812.8576 | 34 | Y11 | 0.0204 | 0.2921 |  | V |
| 112 | Q9UPZ9 | ICK | Serine/threonine-protein kinase ICK | QLGDGTpYGpSVLLGR | 3 | 532.2407 | 26 | Y15,S17 | 0.0178 | 9.2746 | V | V |
| Peptide index | SwissProt no. | Gene name | Protein name | Sequence***^a^*** | Charge state | m/z | Score ***^b^*** | Site | p-value ***^C^*** | Ratio ***^d^*** (SW620/SW480) | PSP | PHOSIDA |
| 113 | Q7Z380 | MTX3 | Putative uncharacterized protein DKFZp686B0395 | pYNADpYELSAK | 2 | 666.7662 | 32 | Y57,Y61 | 0.0174 | 0.4707 |  | X,V |
| 114 | Q6ZWP6 | ACAD8 | Acyl-Coenzyme A dehydrogenase family, member 8, isoform CRA_a | QGDHpYILNGSK | 2 | 656.2900 | 27 | Y56 | 0.0152 | 1.5800 |  | V |
| 115 | Q9HBT7 | ZNF287 | Zinc finger protein 287 | IHSGEKCpYK | 3 | 401.1954 | 25 | Y557 | 0.0132 | 2.1584 |  |  |
| 116 | Q13530 | SERINC3 | Serine incorporator 3 | EMEpTpYLKK | 3 | 401.1782 | 35 | T163,Y64 | 0.0132 | 2.1584 |  | V |
| 117 | Q8N9R8 | SCAI | Protein SCAI | QRSYpYpSQVNK | 2 | 716.8116 | 35 | Y163,S164 | 0.0128 | 38.0202 | V | V |
| 118 | Q9H7J1 | PPP1R3E | Protein phosphatase 1 regulatory subunit 3E | EAPAApYAGPAPPPPR | 4 | 386.4335 | 35 | Y208 | 0.0120 | 10.3829 |  |  |
| 119 | Q15012 | LAPTM4A | Lysosomal-associated transmembrane protein 4A | MPEKEPPPPpYLPA | 3 | 515.5825 | 31 | Y133 | 0.0117 | 0.5485 | V |  |
| Peptide index | SwissProt no. | Gene name | Protein name | Sequence***^a^*** | Charge state | m/z | Score ***^b^*** | Site | p-value ***^C^*** | Ratio ***^d^*** (SW620/SW480) | PSP | PHOSIDA |
| 120 | B1ANS9 | WDR64 | WD repeat-containing protein 64 | FpYASVQK | 2 | 461.7127 | 34 | Y57 | 0.0112 | 1.8278 |  | V |
| 121 | Q4LE39 | ARID4B | AT-rich interactive domain-containing protein 4B | NLDGApYQEAVINK | 2 | 758.3335 | 29 | Y75 | 0.0097 | 0.5040 | V | V |
| 122 | O00264 | PGRMC1 | Membrane-associated progesterone receptor component 1 | EGEEPTVpYSDEEEPK | 2 | 909.3531 | 25 | Y180 | 0.0091 | 0.4488 | V |  |
| 123 | P22607 | FGFR3 | Fibroblast growth factor receptor 3 | DGGEpYLCR | 2 | 496.7147 | 40 | Y607 | 0.0073 | 1.8678 | V |  |
| 124 | E9PAW4 | SORBS2 | Sorbin and SH3 domain-containing protein 2 | MpYSNEDSR | 2 | 541.2242 | 32 | Y2 | 0.0072 | 0.0475 |  |  |
| 125 | Q9UH92 | MLX | Max-like protein X | IMKVNpYEQIVK | 2 | 731.2974 | 40 | Y215 | 0.0071 | 0.5541 | V | V |
| 126 | Q75N90 | FBN3 | Fibrillin-3 | GGTCTNpTDGSpYK | 2 | 710.8400 | 30 | T1090,Y1094 | 0.0067 | 0.0087 |  |  |
| Peptide index | SwissProt no. | Gene name | Protein name | Sequence***^a^*** | Charge state | m/z | Score ***^b^*** | Site | p-value ***^C^*** | Ratio ***^d^*** (SW620/SW480) | PSP | PHOSIDA |
| 127 | P36955 | SERPINF1 | Pigment epithelium-derived factor | pYGLDpSDLSCK | 2 | 630.2725 | 26 | Y253,S257 | 0.0065 | 1.9708 |  |  |
| 128 | Q8NHM5 | KDM2B | Lysine-specific demethylase 2B | KpYCLMpSVK | 3 | 377.8871 | 35 | Y229,S233 | 0.0059 | 2.8195 | V | V |
| 129 | P29353 | SHC1 | SHC-transforming protein 1 | pYMGCVEVLQpSMR | 3 | 531.5832 | 33 | Y166,S175 | 0.0057 | 0.1652 | V |  |
| 130 | Q7Z5J4 | RAI1 | Retinoic acid-induced protein 1 | QPGHpTNYSpSpYSK | 2 | 804.8596 | 28 | T1529,S1533,Y1534 | 0.0052 | 0.2333 | V |  |
| 131 | Q6ZMW3 | EML6 | Echinoderm microtubule-associated protein-like 6 | NNLpYYpTAGK | 2 | 602.3167 | 28 | Y29,T31 | 0.0051 | 7.7559 | V |  |
| 132 | Q6ZMW3 | EML6 | Echinoderm microtubule-associated protein-like 6 | NNLpYYpTAGK | 2 | 602.3167 | 27 | Y29,T31 | 0.0051 | 7.7559 | V |  |
| 133 | Q14247 | CTTN | Src substrate cortactin | TEKHESQRDpYSK | 2 | 794.3500 | 25 | Y302 | 0.0047 | 0.3432 | V |  |
| Peptide index | SwissProt no. | Gene name | Protein name | Sequence***^a^*** | Charge state | m/z | Score ***^b^*** | Site | p-value ***^C^*** | Ratio ***^d^*** (SW620/SW480) | PSP | PHOSIDA |
| 134 | Q8IZT6 | ASPM | AAN40011 | QQpTpYFNKLK | 2 | 665.8404 | 25 | T2207,Y2208 | 0.0046 | 0.3129 | V |  |
| 135 | Q96RT6 | CTAGE1 | Protein cTAGE-2 | NQIpYIQLSGVDK | 3 | 486.5720 | 25 | Y307 | 0.0042 | 14.0851 | V | V |
| 136 | Q96D71 | REPS1 | RalBP1-associated Eps domain-containing protein 1 | QpYpYVNQFK | 2 | 625.7800 | 28 | Y288,Y289 | 0.0040 | 0.5911 | V |  |
| 137 | Q8NFF5 | FLAD1 | FAD synthase | pYNLQMLEAEGSMK | 3 | 532.2376 | 27 | Y456 | 0.0040 | 0.0988 |  |  |
| 138 | P30281 | CCND3 | G1/S-specific cyclin-D3 | ASpYFQCVQR | 2 | 619.3066 | 29 | Y44 | 0.0039 | 0.4123 |  | V |
| 139 | Q53TQ3 | INO80D | INO80 complex subunit D | pYNpSQRCTNPIPK | 3 | 528.5547 | 39 | Y57,S59 | 0.0032 | 0.2294 |  | V |
| 140 | Q9H722 |  | cDNA: FLJ21489 fis, clone COL05450 | EFQKSLGAKHSVpYDpTpTNR | 4 | 581.0094 | 31 | Y45,T47,T48 | 0.0027 | 0.3876 | V | V |

| Peptide index | SwissProt no. | Gene name | Protein name | Sequence***^a^*** | Charge state | m/z | Score ***^b^*** | Site | p-value ***^C^*** | Ratio ***^d^*** (SW620/SW480) | PSP | PHOSIDA |
| --- | --- | --- | --- | --- | --- | --- | --- | --- | --- | --- | --- | --- |
| 141 | Q9NT68 | ODZ2 | Teneurin-2 | ApYNKASGWSVQYR | 2 | 805.8492 | 28 | Y2300 | 0.0026 | 0.2118 | V |  |
| 142 | Q96AC1 | FERMT2 | Fermitin family homolog 2 | LQLPNMKpYVK | 2 | 658.2500 | 26 | Y108 | 0.0026 | 0.2712 | V | V |
| 143 | Q92574 | TSC1 | Tuberous sclerosis 1 | pYLEDVKLQAR | 2 | 658.2619 | 26 | Y929 | 0.0026 | 0.2712 | V |  |
| 144 | Q6N081 | DKFZp686K01114 | Putative uncharacterized protein DKFZp686K01114 | pYERGQNLDLK | 2 | 658.2624 | 33 | Y48 | 0.0026 | 0.2712 |  | V |
| 145 | B4E2K4 |  | cDNA FLJ54576, highly similar to Aspartyl/asparaginyl beta-hydroxylase (EC 1.14.11.16) | pYEGLAVMDK | 2 | 552.7569 | 25 | Y570 | 0.0022 | 0.5765 |  | V |
| 146 | Q6P534 | TPO | TPO protein | pYRPIpTGACNNRK | 2 | 804.8596 | 37 | Y151,T155 | 0.0021 | 73.7585 |  | V |

| Peptide index | SwissProt no. | Gene name | Protein name | Sequence***^a^*** | Charge state | m/z | Score ***^b^*** | Site | p-value ***^C^*** | Ratio ***^d^*** (SW620/SW480) | PSP | PHOSIDA |
| --- | --- | --- | --- | --- | --- | --- | --- | --- | --- | --- | --- | --- |
| 147 | Q92535 | PIGC | Phosphatidylinositol N-acetylglucosaminyltransferase subunit C | pYAQPVpTNpTK | 2 | 631.8023 | 28 | Y2,T7,T9 | 0.0019 | 0.3461 |  |  |
| 148 | O15394 | NCAM2 | Neural cell adhesion molecule 2 | VAAVNGKGQGDpYSK | 2 | 737.3252 | 33 | Y580 | 0.0017 | 0.1013 |  | V |
| 149 | A8MT65 | ZNF891 | Zinc finger protein 891 | SFGpTpSSpYLIVHK | 2 | 789.3390 | 29 | T415,S416,Y418 | 0.0015 | 0.1243 |  | V |
| 150 | O43526 | KCNQ2 | Potassium voltage-gated channel subfamily KQT member 2 | SLSApYGGGNR | 2 | 531.2592 | 46 | Y755 | 0.0015 | 0.2874 | V |  |
| 151 | Q9NZQ9 | TMOD4 | Tropomodulin-4 | MpSSpYQKELEK | 3 | 473.1977 | 25 | S2,Y4 | 0.0014 | 0.1604 |  |  |
| 152 | Q9NR48 | ASH1L | Histone-lysine N-methyltransferase ASH1L | KAYpYNAR | 2 | 482.7172 | 34 | Y2544 | 0.0014 | 5.4046 | V | V |
| 153 | O75128 | COBL | Protein cordon-bleu | pTSpSQpYVASAIAKR | 3 | 541.2535 | 25 | T863,S865,Y867 | 0.0011 | 0.2928 | V | V |
| Peptide index | SwissProt no. | Gene name | Protein name | Sequence***^a^*** | Charge state | m/z | Score ***^b^*** | Site | p-value ***^C^*** | Ratio ***^d^*** (SW620/SW480) | PSP | PHOSIDA |
| 154 | Q7Z4N2 | TRPM1 | Transient receptor potential cation channel subfamily M member 1 | ITRpYFPDEpTINACKTMK | 3 | 730.6967 | 30 | Y1423,T1428 | 0.0010 | 0.2660 |  |  |
| 155 | P49840 | GSK3A | Glycogen synthase kinase-3 alpha | GEPNVSpYICSR | 2 | 681.2810 | 29 | Y279 | 0.0009 | 0.4910 | V |  |
| 156 | Q96P65 | QRFPR | Pyroglutamylated RFamide peptide receptor | KNVLpSAVCpYCIVNK | 3 | 571.9538 | 30 | S345,Y349 | 0.0008 | 4.9180 |  | V |
| 157 | Q08752 | PPID | Peptidyl-prolyl cis-trans isomerase D | pYVDSpSKAVIETADR | 3 | 571.9538 | 29 | Y252,S256 | 0.0008 | 4.9180 | V |  |
| 158 | Q13621 | SLC12A1 | Solute carrier family 12 member 1 | MKPNTLVIGpYK | 3 | 454.2248 | 39 | Y781 | 0.0008 | 0.5297 | V |  |
| 159 | Q8NH60 | OR52J3 | Olfactory receptor 52J3 | VLpYVFpTK | 2 | 514.7400 | 27 | Y306,T309 | 0.0007 | 10.3514 | V |  |
| 160 | Q9NYY3 | PLK2 | Serine/threonine-protein kinase PLK2 | TIpTpYQPAASpTK | 3 | 473.8580 | 25 | T8,Y9,T15 | 0.0007 | 0.2166 | V |  |

| Peptide index | SwissProt no. | Gene name | Protein name | Sequence***^a^*** | Charge state | m/z | Score ***^b^*** | Site | p-value ***^C^*** | Ratio ***^d^*** (SW620/SW480) | PSP | PHOSIDA |
| --- | --- | --- | --- | --- | --- | --- | --- | --- | --- | --- | --- | --- |
| 161 | Q8NGM6 |  | Seven transmembrane helix receptor | pYTTILTpSMVNGK | 2 | 743.8538 | 35 | Y143,S149 | 0.0007 | 0.5618 |  | V |
| 162 | Q15652 | JMJD1C | Probable JmjC domain-containing histone demethylation protein 2C | VGSTDAGIAFAPVpYAMGAPSSK | 3 | 731.3468 | 26 | Y2092 | 0.0006 | 3.3812 |  |  |
| 163 | Q8TE01 | derp12 | DERP12 (Dermal papilla derived protein 12) | NIKIpYENSKVVK | 2 | 758.3002 | 29 | Y233 | 0.0006 | 5.3313 |  |  |
| 164 | Q3KR37 | GRAMD1B | GRAM domain-containing protein 1B | DpYTSESEEK | 3 | 389.8176 | 36 | Y726 | 0.0005 | 0.3916 | V |  |
| 165 | Q8HWG5 | HLA-DRB1 | MHC class II antigen | pYFYNQEEpYAR | 2 | 771.8222 | 31 | Y25,Y32 | 0.0005 | 2.0002 |  |  |
| 166 | Q08426 | EHHADH | Peroxisomal bifunctional enzyme | GWpYQpYDKPLGR | 2 | 771.8500 | 46 | Y580,Y582 | 0.0005 | 2.0002 |  |  |
| 167 | Q96M11 | HYLS1 | Hydrolethalus syndrome protein 1 | EAQpSIQpYDPYSK | 2 | 794.8470 | 39 | S48,Y51 | 0.0004 | 0.2541 | V |  |

| Peptide index | SwissProt no. | Gene name | Protein name | Sequence***^a^*** | Charge state | m/z | Score ***^b^*** | Site | p-value ***^C^*** | Ratio ***^d^*** (SW620/SW480) | PSP | PHOSIDA |
| --- | --- | --- | --- | --- | --- | --- | --- | --- | --- | --- | --- | --- |
| 168 | Q9C0C4 | SEMA4C | Semaphorin-4C | NpSNANGpYVR | 2 | 578.2736 | 33 | S784,Y789 | 0.0003 | 3.5011 |  | V |
| 169 | Q9C0C4 | SEMA4C | Semaphorin-4C | NpSNANGpYVR | 2 | 578.2736 | 27 | S784,Y789 | 0.0003 | 3.5011 |  | V |
| 170 | O14686 | MLL2 | Histone-lysine N-methyltransferase MLL2 | EPEPQpYFR | 2 | 573.2400 | 27 | Y1584 | 0.0003 | 8.1737 | V |  |
| 171 | Q06278 | AOX1 | Aldehyde oxidase | pYGCGGGGCGACTVMISR | 3 | 582.2700 | 33 | Y42 | 0.0003 | 24.2226 | V |  |
| 172 | Q9Y227 | ENTPD4 | Ectonucleoside triphosphate diphosphohydrolase 4 | MGGDpYNAAK | 2 | 512.2409 | 25 | Y448 | 0.0003 | 0.3074 | V |  |
| 173 | Q9Y6Y1 | CAMTA1 | Calmodulin-binding transcription activator 1 | QGQTpYGGGGLK | 2 | 574.2222 | 30 | Y498 | 0.0002 | 0.1413 | V |  |
| 174 | Q14593 | ZNF273 | Zinc finger protein 273 | IHpTGEKPpYKPK | 3 | 486.5349 | 27 | T537,Y542 | 0.0002 | 34.6716 |  |  |

| Peptide index | SwissProt no. | Gene name | Protein name | Sequence***^a^*** | Charge state | m/z | Score ***^b^*** | Site | p-value ***^C^*** | Ratio ***^d^*** (SW620/SW480) | PSP | PHOSIDA |
| --- | --- | --- | --- | --- | --- | --- | --- | --- | --- | --- | --- | --- |
| 175 | Q96N16 | JAKMIP1 | Janus kinase and microtubule-interacting protein 1 | FCQLTREpYQALQR | 3 | 579.2752 | 29 | Y495 | 0.0001 | 12.2317 |  | V |
| 176 | Q9H4L2 | BRCA2 | Breast cancer 2 tumor suppressor | KTQpYQQLP | 2 | 544.2747 | 25 | Y50 | 0.0001 | 0.1571 | V |  |
| 177 | P31273 | HOXC8 | Homeobox protein Hox-C8 | MSpSpYFVNPLFSK | 2 | 798.3444 | 29 | S3,Y4 | 9.70E-05 | 0.1094 |  |  |
| 178 | Q6ZNI2 |  | cDNA FLJ16039 fis, clone ADRGL2001554, weakly similar to SERINE/THREONINE-PROTEIN KINASE PLK | pYITELDETKpTK | 2 | 750.3400 | 37 | Y24,T33 | 8.19E-05 | 0.1962 |  | V |
| 179 | Q6P1N0 | CC2D1A | Coiled-coil and C2 domain-containing protein 1A | EpYpTAQLER | 3 | 390.2102 | 41 | Y95,T96 | 3.43E-05 | 0.3718 | V |  |

| Peptide index | SwissProt no. | Gene name | Protein name | Sequence***^a^*** | Charge state | m/z | Score ***^b^*** | Site | p-value ***^C^*** | Ratio ***^d^*** (SW620/SW480) | PSP | PHOSIDA |
| --- | --- | --- | --- | --- | --- | --- | --- | --- | --- | --- | --- | --- |
| 180 | Q7Z5R6 | APBB1IP | Amyloid beta A4 precursor protein-binding family B member 1-interacting protein | pYAVFKNPQNFYLDNR | 3 | 657.6054 | 36 | Y264 | 2.91E-05 | 0.1405 | V |  |
| 181 | O15399 | GRIN2D | Glutamate [NMDA] receptor subunit epsilon-4 | pYYGPIEPQGLGLGLGEAR | 3 | 657.6046 | 35 | Y964 | 2.91E-05 | 0.1405 |  |  |
| 182 | Q709C8 | VPS13C | Vacuolar protein sorting-associated protein 13C | CPANNMEpYLVGVSIK | 3 | 579.2600 | 28 | Y2849 | 2.69E-05 | 7.3934 | V |  |
| 183 | P16615 | ATP2A2 | Sarcoplasmic/endoplasmic reticulum calcium ATPase 2 | KSMSVpYCTPNKPSR | 3 | 579.2381 | 25 | Y497 | 2.69E-05 | 7.3934 | V | V |
| 184 | B4DIZ5 | Adenosylhomocysteinase | Adenosylhomocysteinase | KpYIVNGNpSGIK | 3 | 452.1767 | 30 | Y8,S14 | 1.51E-05 | 0.0444 |  |  |
| 185 | Q7Z692 | CEACAM19 | Carcinoembryonic antigen-related cell adhesion molecule 19 | LQGSQAALpYIQK | 2 | 701.2720 | 29 | Y35 | 1.44E-05 | 133.7159 |  | V |

| Peptide index | SwissProt no. | Gene name | Protein name | Sequence***^a^*** | Charge state | m/z | Score ***^b^*** | Site | p-value ***^C^*** | Ratio ***^d^*** (SW620/SW480) | PSP | PHOSIDA |
| --- | --- | --- | --- | --- | --- | --- | --- | --- | --- | --- | --- | --- |
| 186 | Q86X24 | HORMAD1 | HORMA domain-containing protein 1 | pYTNNGPLMDFISK | 2 | 798.8400 | 25 | Y121 | 1.31E-05 | 0.2637 |  |  |
| 187 | P82094 | TMF1 | TATA element modulatory factor | pSIQAALDpSApYK | 3 | 469.2136 | 25 | S655,S662,Y664 | 1.23E-05 | 0.2853 | V | V |
| 188 | Q9Y6X0 | SETBP1 | SET-binding protein | pYSGSGGDGGpSTR | 2 | 630.2873 | 28 | Y1263,S1272 | 8.86E-06 | 0.1416 | V |  |
| 189 | Q8IYL2 | METTL19 | Probable tRNA (uracil-O(2)-)-methyltransferase | MSNVpYQIQLSHSK | 3 | 544.2264 | 27 | Y234 | 6.42E-06 | 0.1325 |  |  |
| 190 | Q9P275 | USP36 | Ubiquitin carboxyl-terminal hydrolase 36 | pYTIDAMQKACLNGCAKLDR | 3 | 737.7060 | 33 | Y215 | 2.63E-06 | 0.2128 | V |  |
| 191 | Q8WVS4 | WDR60 | WD repeat-containing protein 60 | pYSKEKpSNSFSDK | 2 | 789.8877 | 25 | Y240,S245 | 2.15E-06 | 27.8203 |  |  |
| 192 | Q86X24 | HORMAD1 | HORMA domain-containing protein 1 | pYTNNGPLMDFISK | 2 | 799.3403 | 32 | Y101 | 1.97E-06 | 0.1566 |  | V |

| Peptide index | SwissProt no. | Gene name | Protein name | Sequence***^a^*** | Charge state | m/z | Score ***^b^*** | Site | p-value ***^C^*** | Ratio ***^d^*** (SW620/SW480) | PSP | PHOSIDA |
| --- | --- | --- | --- | --- | --- | --- | --- | --- | --- | --- | --- | --- |
| 193 | Q7Z5R6 | APBB1IP | Amyloid beta A4 precursor protein-binding family B member 1-interacting protein | pYAVFKNPQNFYLDNR | 3 | 657.2685 | 36 | Y264 | 1.47E-06 | 0.1439 | V |  |
| 194 | B4DMX4 |  | cDNA FLJ57154, highly similar to Alpha-fetoprotein | NKFIpYEIAR | 2 | 617.3000 | 26 | Y6 | 1.16E-06 | 0.0793 |  |  |
| 195 | Q8IYB5 | SMAP1 | Stromal membrane-associated protein 1 | pYYDKNAIAITNKEK | 3 | 584.6070 | 37 | Y128 | 7.59E-07 | 177.9859 |  |  |
| 196 | P36639 | NUDT1 | 7,8-dihydro-8-oxoguanine triphosphatase | MpYWpSNQITR | 3 | 453.2440 | 25 | Y2,S4 | 2.94E-07 | 0.3064 |  |  |
| 197 | Q9C0C2 | TNKS1BP1 | 182 kDa tankyrase-1-binding protein | DSQGTpYSSR | 2 | 540.7332 | 32 | Y855 | 2.80E-07 | 0.0906 | V |  |
| 198 | Q86W56 | PARG | Poly(ADP-ribose) glycohydrolase | pYLDQFVPEK | 2 | 610.3113 | 49 | Y832 | 8.90E-09 | 0.0557 |  |  |
| Peptide index | SwissProt no. | Gene name | Protein name | Sequence***^a^*** | Charge state | m/z | Score ***^b^*** | Site | p-value ***^C^*** | Ratio ***^d^*** (SW620/SW480) | PSP | PHOSIDA |
| 199 | Q6P2C8 | MED27 | Mediator of RNA polymerase II transcription subunit 27 | LQpYHAGLASGLLNQQpSLK | 3 | 701.3139 | 27 | Y119,S132 | 1.21E-14 | 10.4028 |  |  |
| 200 | Q9Y6M1 | IGF2BP2 | Insulin-like growth factor 2 mRNA-binding protein 2 | pYPQGVASQR | 2 | 543.7224 | 30 | Y589 |  |  | V | V |
| 201 | Q9Y6M1 | IGF2BP2 | Insulin-like growth factor 2 mRNA-binding protein 2 | pYPQGVASQR | 2 | 544.2227 | 29 | Y589 |  |  | V | V |
| 202 | Q9Y664 | KPTN | Kaptin | FSpSQNNVpYGLAGGAGGR | 3 | 605.7488 | 34 | S24,Y29 |  |  |  |  |
| 203 | Q9Y5T4 | DNAJC15 | DnaJ homolog subfamily C member 15 | RVMILNHPDKGGpSPpYVAAK | 3 | 743.9600 | 33 | S130,Y132 |  |  |  |  |
| 204 | Q9Y4G6 | TLN2 | Talin-2 | NGDILEpYK | 2 | 516.2300 | 33 | Y83 |  |  | V |  |
| 205 | Q9Y2K3 | MYH15 | Myosin-15 | EVMAApYK | 2 | 454.2172 | 30 | Y160 |  |  | V |  |

| Peptide index | SwissProt no. | Gene name | Protein name | Sequence***^a^*** | Charge state | m/z | Score ***^b^*** | Site | p-value ***^C^*** | Ratio ***^d^*** (SW620/SW480) | PSP | PHOSIDA |
| --- | --- | --- | --- | --- | --- | --- | --- | --- | --- | --- | --- | --- |
| 206 | Q9ULX3 | NOB1 | RNA-binding protein NOB1 | pYSLPpTPKGGK | 2 | 604.3536 | 25 | Y324,T328 |  |  |  |  |
| 207 | Q9ULI3 | HEG1 | Protein HEG homolog 1 | SpYSESSSpTSSSESLNSSAPR | 3 | 737.3276 | 27 | Y507 |  |  | V | V |
| 208 | Q9ULH0 | KIDINS220 | Kinase D-interacting substrate of 220 kDa | EpYIAQMSQLEGGPGSpTpTISGR | 4 | 606.5700 | 28 | Y1390,T1404,T1405 |  |  |  |  |
| 209 | Q9UKY4 | POMT2 | Protein O-mannosyl-transferase 2 | MGSpYYINR | 2 | 542.5800 | 32 | Y95 |  |  |  | V |
| 210 | Q9P2P5 | HECW2 | E3 ubiquitin-protein ligase HECW2 | pYQSIRR | 2 | 452.5000 | 42 | Y563 |  |  |  | V |
| 211 | Q9NV66 | TYW1 | tRNA wybutosine-synthesizing protein 1 homolog | TFpSAKDpYMAR | 3 | 455.7341 | 29 | S692,Y696 |  |  |  |  |
| 212 | Q9NRM7 | LATS2 | Serine/threonine-protein kinase LATS2 | pYpSLLPFANESGTpSAAAEVNR | 4 | 585.1963 | 27 | Y82,S83,S94 |  |  | V | V |
| Peptide index | SwissProt no. | Gene name | Protein name | Sequence***^a^*** | Charge state | m/z | Score ***^b^*** | Site | p-value ***^C^*** | Ratio ***^d^*** (SW620/SW480) | PSP | PHOSIDA |
| 213 | Q9HBL0 | TNS1 | Tensin-1 | EKQGAMpYHTQHLR | 3 | 566.2958 | 28 | Y458 |  |  |  |  |
| 214 | Q9H7T9 | C1orf135 | Uncharacterized protein C1orf135 | ENRQAPVLLQpTpYR | 4 | 438.5439 | 27 | T249,Y250 |  |  |  |  |
| 215 | Q9H0D6 | XRN2 | 5'-3' exoribonuclease 2 | pYPSIIVNCVEEK | 3 | 491.7093 | 25 | Y14 |  |  | V | V |
| 216 | Q9H0D2 | ZNF541 | Zinc finger protein 541 | NCSQMFpYTEK | 2 | 694.7593 | 32 | Y848 |  |  |  |  |
| 217 | Q9C0B9 | ZCCHC2 | Zinc finger CCHC domain-containing protein 2 | QpSSMEANQQGTpYR | 4 | 416.5200 | 31 | S1149,Y1159 |  |  |  |  |
| 218 | Q9BZE3 | BARHL1 | BarH-like 1 homeobox protein | RQTAVGLELLAEAGNpYSALQR | 4 | 606.6132 | 27 | Y250 |  |  |  | V |

| Peptide index | SwissProt no. | Gene name | Protein name | Sequence***^a^*** | Charge state | m/z | Score ***^b^*** | Site | p-value ***^C^*** | Ratio ***^d^*** (SW620/SW480) | PSP | PHOSIDA |
| --- | --- | --- | --- | --- | --- | --- | --- | --- | --- | --- | --- | --- |
| 219 | Q9BY15 | EMR3 | EGF-like module-containing mucin-like hormone receptor-like 3 | SEpSEpTpYpTLSSK | 2 | 816.1700 | 31 | S623,T625,Y626,T627 |  |  |  |  |
| 220 | Q9BWM7 | SFXN3 | Sideroflexin-3 | QLGpTApYVSATpTGAVApTALGLK | 3 | 771.8500 | 26 | T152,Y154,T159,T164 |  |  |  |  |
| 221 | Q9BQ13 | KCTD14 | BTB/POZ domain-containing protein KCTD14 | QFLLQVPGpYpSENLELMVR | 3 | 771.8500 | 33 | Y145,S146 |  |  |  |  |
| 222 | Q9BPW8 | NIPSNAP1 | Protein NipSnap homolog 1 | FSGGpYPALMDCMNK | 2 | 816.1700 | 32 | Y132 |  |  | V | V |
| 223 | Q96QD5 | DEPDC7 | DEP domain-containing protein 7 | DpYSNNTEKTTK | 2 | 690.3100 | 29 | Y460 |  |  |  |  |
| 224 | Q96JF4 | KIAA1873 | KIAA1873 protein | AGpYVQEEQK | 3 | 378.6400 | 31 | Y165 |  |  | V | V |

| Peptide index | SwissProt no. | Gene name | Protein name | Sequence***^a^*** | Charge state | m/z | Score ***^b^*** | Site | p-value ***^C^*** | Ratio ***^d^*** (SW620/SW480) | PSP | PHOSIDA |
| --- | --- | --- | --- | --- | --- | --- | --- | --- | --- | --- | --- | --- |
| 225 | Q96FC9 | DDX11 | Probable ATP-dependent RNA helicase DDX11 | pYGAVFpSpSREQPK | 2 | 805.4412 | 48 | Y515,S520,S521 |  |  | V | V |
| 226 | Q969G2 | LHX4 | LIM/homeobox protein Lhx4 | LVCKEDpYETAK | 2 | 690.2900 | 30 | Y144 |  |  |  | V |
| 227 | Q92574 | TSC1 | Tuberous sclerosis 1 | pYNQLQEQRDTMVTKLHSQIR | 3 | 857.4151 | 25 | Y761 |  |  | V | V |
| 228 | Q92535 | PIGC | Phosphatidylinositol N-acetylglucosaminyltransferase subunit C | MpYAQPVpTNTKEVK | 2 | 843.3838 | 26 | Y2,T7 |  |  |  |  |
| 229 | Q8TE77 | SSH3 | Protein phosphatase Slingshot homolog 3 | QLQIpYQGILpTApSR | 3 | 578.6172 | 27 | Y463,T468,S470 |  |  |  | V |
| 230 | Q8N5D0 | WDTC1 | BAA82989 WD and tetratricopeptide repeats protein 1 | LPDpYNNR | 2 | 486.5300 | 33 | Y262 |  |  |  | V |

| Peptide index | SwissProt no. | Gene name | Protein name | Sequence***^a^*** | Charge state | m/z | Score ***^b^*** | Site | p-value ***^C^*** | Ratio ***^d^*** (SW620/SW480) | PSP | PHOSIDA |
| --- | --- | --- | --- | --- | --- | --- | --- | --- | --- | --- | --- | --- |
| 231 | Q8IYM0 | FAM186B | Protein FAM186B | QRPMSpSVEFpTpYRPRTRR | 4 | 606.5345 | 28 | S613,T617,Y618 |  |  | V |  |
| 232 | Q8IUX1 | TMEM126B | Transmembrane protein 126B | NFDpYLR | 2 | 454.8674 | 33 | Y63 |  |  |  |  |
| 233 | Q86W56 | PARG | Poly(ADP-ribose) glycohydrolase | KTQpYQQLP | 2 | 544.4500 | 31 | Y832 |  |  |  |  |
| 234 | Q86TV6 | TTC7B | Tetratricopeptide repeat protein 7B | VGLDDLPLpTAVPPpYR | 3 | 595.9875 | 31 | T131,Y136 |  |  |  |  |
| 235 | Q7Z7G8 | VPS13B | Vacuolar protein sorting-associated protein 13B | SWpYHGQTpSMPGTLVLCLPQIK | 2 | 846.3700 | 33 | Y1104,S1109 |  |  | V |  |
| 236 | Q7Z5L9 | IRF2BP2 | Interferon regulatory factor 2-binding protein 2 | QSIKQQGASGEVpYCPSGEK | 3 | 738.0346 | 30 | S537,Y548 |  |  |  |  |
| 237 | Q7Z3S7 | CACNA2D4 | Voltage-dependent calcium channel subunit alpha-2/delta-4 | pYSGSLLLQK | 2 | 585.2029 | 35 | Y95 |  |  | V |  |
| Peptide index | SwissProt no. | Gene name | Protein name | Sequence***^a^*** | Charge state | m/z | Score ***^b^*** | Site | p-value ***^C^*** | Ratio ***^d^*** (SW620/SW480) | PSP | PHOSIDA |
| 238 | Q7KYZ0 |  | p65 | SQGNpTDYpYCFpTNDCNISK | 3 | 771.8277 | 26 | T72,Y75,T78 |  |  |  |  |
| 239 | Q6ZSB9 | ZBTB49 | Zinc finger and BTB domain-containing protein 49 | ApSpSpTTpYRNSEGQFFSSMpTLWGLAMK |  | 469.7258 | 28 | S732,S733,T734,Y736,T748 |  |  | V |  |
| 240 | Q6ZNI2 |  | cDNA FLJ16039 fis, clone ADRGL2001554, weakly similar to SERINE/THREONINE-PROTEIN KINASE PLK | pYRVGKQIGK | 2 | 564.5800 | 26 | Y39 |  |  |  |  |
| 241 | Q6XPR3 | RPTN | Repetin | QDQSpYHYGQTDR | 4 | 415.9154 | 32 | Y305 |  |  | V | V |
| 242 | Q6DN90 | IQSEC1 | IQ motif and SEC7 domain-containing protein 1 | QSApYERSLGGQQGSP | 2 | 887.9511 | 32 | Y407 |  |  | V | V |
| 243 | Q68CQ4 | DIEXF | Digestive organ expansion factor homolog | ELFLIMNpSpYR | 3 | 482.7000 | 27 | S288,Y289 |  |  |  | V |
| Peptide index | SwissProt no. | Gene name | Protein name | Sequence***^a^*** | Charge state | m/z | Score ***^b^*** | Site | p-value ***^C^*** | Ratio ***^d^*** (SW620/SW480) | PSP | PHOSIDA |
| 244 | Q5VW32 | BROX | BRO1 domain-containing protein BROX | ATAPVpSFNYpYGVVTGPSASK | 3 | 726.3000 | 29 | S17,Y21 |  |  | V | V |
| 245 | Q5THR3 | EFCAB6 | EF-hand calcium-binding domain-containing protein 6 | pYILNCMAVK | 2 | 568.6300 | 27 | Y430 |  |  | V |  |
| 246 | Q5THK1 | PRR14L | Protein PRR14L | SpSCKVpSpYpTSQER | 4 | 438.5062 | 29 | S846,S850,Y851,T852 |  |  | V |  |
| 247 | Q5TCW0 | C9orf127 | Chromosome 9 open reading frame 127 | VFVPpSFTpYR | 3 | 425.7200 | 40 | S264,Y267 |  |  |  |  |
| 248 | Q5T7N3 | KANK4 | KN motif and ankyrin repeat domain-containing protein 4 | NGNpTALHpYSVSHSNFSIAK | 3 | 737.2900 | 28 | T826,Y830 |  |  |  |  |
| 249 | Q5JTA8 | RP11-217H1.1 | Novel protein | TKNpYLNK | 2 | 480.8700 | 27 | Y234 |  |  |  | V |

| Peptide index | SwissProt no. | Gene name | Protein name | Sequence***^a^*** | Charge state | m/z | Score ***^b^*** | Site | p-value ***^C^*** | Ratio ***^d^*** (SW620/SW480) | PSP | PHOSIDA |
| --- | --- | --- | --- | --- | --- | --- | --- | --- | --- | --- | --- | --- |
| 250 | Q59G12 |  | Lamina-associated polypeptide 2, isoforms beta/gamma variant | pYGVNPGPIVGTTR | 2 | 705.5800 | 25 | Y160 |  |  | V |  |
| 251 | Q32P41 | TRMT5 | tRNA (guanine-N(1)-)-methyltransferase | INNIDNMpYR | 2 | 617.2917 | 28 | Y247 |  |  |  |  |
| 252 | Q16880 | UGT8 | 2-hydroxyacylsphingosine 1-beta-galactosyltransferase | pYLpSEDIANK | 2 | 606.5700 | 28 | Y299,S301 |  |  |  |  |
| 253 | Q16706 | MAN2A1 | Alpha-mannosidase 2 | KVpYNREIAMK | 3 | 449.8632 | 26 | Y874 |  |  |  |  |
| 254 | Q15911 | ZFHX3 | Zinc finger homeobox protein 3 | pYQQpTLEAHMK | 2 | 705.5800 | 28 | Y682,685 |  |  |  |  |
| 255 | Q15788 | NCOA1 | Nuclear receptor coactivator 1 | pYEVMQCFTVpSQPK | 3 | 579.2270 | 25 | Y218,S227 |  |  |  | V |
| 256 | Q15149 | PLEC | Plectin | pYASGSpSASLGGPESAVA | 3 | 557.7400 | 26 | Y4668,S4673 |  |  | V |  |
| Peptide index | SwissProt no. | Gene name | Protein name | Sequence***^a^*** | Charge state | m/z | Score ***^b^*** | Site | p-value ***^C^*** | Ratio ***^d^*** (SW620/SW480) | PSP | PHOSIDA |
| 257 | Q15020 | SART3 | Squamous cell carcinoma antigen recognized by T-cells 3 | pYSTSLEK | 2 | 454.5318 | 30 | Y794 |  |  | V |  |
| 258 | Q14993 | COL19A1 | Collagen alpha-1(XIX) chain | GEEGGAGEPGKpYDSMAR | 3 | 602.7800 | 28 | Y722 |  |  | V | V |
| 259 | Q14524 | SCN5A | Sodium channel protein type 5 subunit alpha | KTIKVLLEpYADK | 2 | 750.6300 | 25 | Y1241 |  |  |  | V |
| 260 | Q13099 | IFT88 | Intraflagellar transport protein 88 homolog | DSRVKSAAATNLpSALpYYMGK | 4 | 581.2547 | 31 | S466,Y469 |  |  | V |  |
| 261 | Q07617 | SPAG1 | Sperm-associated antigen 1 | RATpTpYK | 2 | 449.9114 | 27 | T285,Y286 |  |  | V |  |
| 262 | P78559 | MAP1A | Microtubule-associated protein 1A | ASYpYVVpSGNDPANGEPSR | 3 | 681.3341 | 28 | Y2720,S2723 |  |  | V | V |
| 263 | P59095 | STARD6 | StAR-related lipid transfer protein 6 | AIAQQTAQEVLGpYNR | 3 | 581.9296 | 25 | Y17 |  |  |  |  |

| Peptide index | SwissProt no. | Gene name | Protein name | Sequence***^a^*** | Charge state | m/z | Score ***^b^*** | Site | p-value ***^C^*** | Ratio ***^d^*** (SW620/SW480) | PSP | PHOSIDA |
| --- | --- | --- | --- | --- | --- | --- | --- | --- | --- | --- | --- | --- |
| 264 | P55199 | ELL | RNA polymerase II elongation factor ELL | KpTNTNpYSQEK | 2 | 687.9500 | 28 | T584,Y588 |  |  |  |  |
| 265 | P54105 | CLNS1A | Methylosome subunit pICln | LEGMLSQpSVpSSQpYNMAGVR | 3 | 771.8500 | 31 | S195,S197,Y200 |  |  | V |  |
| 266 | P51843 | NR0B1 | Nuclear receptor subfamily 0 group B member 1 | NVALLpYRCCFCGK | 3 | 524.2790 | 30 | Y64 |  |  |  |  |
| 267 | P29074 | PTPN4 | Tyrosine-protein phosphatase non-receptor type 4 | pYQpYFLQIK | 2 | 632.2500 | 29 | Y124,Y126 |  |  | V |  |
| 268 | P26718 | KLRK1 | NKG2-D type II integral membrane protein | GYIENCSTPNpTpYICMQR | 3 | 743.0379 | 28 | T197,Y198 |  |  |  |  |
| 269 | P25391 | LAMA1 | Laminin subunit alpha-1 | CSSSYpYGNPQTPGGSCQK | 3 | 648.6435 | 30 | Y836 |  |  | V |  |
| 270 | P20815 | CYP3A5 | Cytochrome P450 3A5 | FDTECpYK | 2 | 493.5500 | 25 | Y65 |  |  | V | V |
| Peptide index | SwissProt no. | Gene name | Protein name | Sequence***^a^*** | Charge state | m/z | Score ***^b^*** | Site | p-value ***^C^*** | Ratio ***^d^*** (SW620/SW480) | PSP | PHOSIDA |
| 271 | P07998 | RNASE1 | Ribonuclease pancreatic | pYPNCAYRTSPK | 2 | 690.8779 | 32 | Y120 |  |  |  | V |
| 272 | P04275 | VWF | von Willebrand factor | pYDVCpSCSDGR | 2 | 632.6600 | 25 | Y610,S614 |  |  |  |  |
| 273 | O95644 | NFATC1 | Nuclear factor of activated T-cells, cytoplasmic 1 | RSQpYQR | 2 | 459.9055 | 26 | Y688 |  |  | V |  |
| 274 | O00429 | DNM1L | Dynamin-1-like protein | pYLARTLNR | 2 | 544.3953 | 36 | Y284 |  |  |  |  |
| 275 | B4E2T6 |  | cDNA FLJ58231, highly similar to NMDA receptor-regulated protein 1 | MRDLEGpYR | 2 | 568.6300 | 33 | Y7 |  |  | V | V |
| 276 | B4DZU5 | TAF8 | Uncharacterized protein | MLQSpYIpSEIGR | 2 | 737.2800 | 29 | Y5,S7 |  |  | V |  |
| 277 | B3KXM2 |  | Serine/threonine-protein phosphatase | pYGQFSGLNPGG | 2 | 589.0319 | 29 | Y403 |  |  |  |  |
| Peptide index | SwissProt no. | Gene name | Protein name | Sequence***^a^*** | Charge state | m/z | Score ***^b^*** | Site | p-value ***^C^*** | Ratio ***^d^*** (SW620/SW480) | PSP | PHOSIDA |
| 278 | B2R4X7 | HMGB4 | Uncharacterized protein | pYRMSARNR | 3 | 378.6400 | 25 | Y170 |  |  |  |  |
| 279 | A8K1T6 |  | cDNA FLJ77270, highly similar to Homo sapiens methyltransferase like 4 (METTL4), mRNA | SSFLLSGIpSCMQPLLNpYR | 4 | 566.7589 | 32 | S269,Y277 |  |  |  |  |
| 280 | A6NI61 | TMEM8C | Transmembrane protein 8C | WGpYGVYpSGPIGTAILIIAAK | 3 | 737.2800 | 28 | Y115,S119 |  |  |  |  |

^a^ modified residues were underlined, indicating phosphorylation (STY), oxidation (M), carboxyamidomethylation (C), and deamidation (N,Q)

^b^ Score derived from the mascot

^c^ p-value refers to the significance or the difference between SW480 and SW620 cells by using one-way ANOVA

^d^ ratio derived from the normalized peak abundance from the SW620 sample divided by that of the SW480 sample,

Abbreviations:

PSP: PhosphoSitePlus (http://www.phosphosite.org.)

PHOSIDA: phosphorylation site database (http://www.phosida.com/)

The “p” indicates a phosphate group on the following residue.
